# The protein phosphatase PPKL is a key regulator of daughter parasite development in *Toxoplasma gondii*

**DOI:** 10.1128/mbio.02254-23

**Published:** 2023-10-25

**Authors:** Chunlin Yang, Emma H. Doud, Emily Sampson, Gustavo Arrizabalaga

**Affiliations:** 1 Department of Pharmacology and Toxicology, Indiana University School of Medicine, Indianapolis, Indiana, USA; 2 Department of Biochemistry and Molecular Biology, Indiana University School of Medicine, Indianapolis, Indiana, USA; 3 Center for Proteome Analysis, Indiana University School of Medicine, Indianapolis, Indiana, USA; 4 Melvin and Bren Simon Comprehensive Cancer Center, Indiana University School of Medicine, Indianapolis, Indiana, USA; 5 Department of Microbiology and Immunology, Indiana University School of Medicine, Indianapolis, Indiana, USA; UT Southwestern Medical Center, Dallas, Texas, USA

**Keywords:** *Toxoplasma*, phosphatase, PPKL, DYRK1, SPM1, Crk1, division, cell cycle, phosphorylation

## Abstract

Apicomplexan parasites, including *Toxoplasma gondii*, encode many plant-like proteins, which play significant roles and present attractive targets for drug development. In this study, we have characterized the plant-like protein phosphatase PPKL, which is unique to the parasite and absent in its mammalian host. We have shown that its localization changes as the parasite divides. In non-dividing parasites, it is present in the cytoplasm, nucleus, and preconoidal region. As the parasite begins division, PPKL is enriched in the preconoidal region and the cortical cytoskeleton of nascent parasites. Later in the division, PPKL is present in the basal complex ring. Conditional knockdown of PPKL showed that it is essential for parasite propagation. Moreover, parasites lacking PPKL exhibit uncoupling of division, with normal DNA duplication but severe defects in forming daughter parasites. While PPKL depletion does not impair the duplication of centrosomes, it affects the stability of cortical microtubules. Both co-immunoprecipitation and proximity labeling identified the kinase DYRK1 as a potential functional partner of PPKL. Complete knockout of *DYRK1* causes parasites to exhibit division defects with predominantly asynchronous divisions. Global phosphoproteomics analysis revealed a significant increase in phosphorylation of the microtubule-associated protein SPM1 in PPKL-depleted parasites, suggesting that PPKL regulates cortical microtubules by mediating the phosphorylation state of SPM1. More importantly, the phosphorylation of cell cycle-associated kinase Crk1, a known regulator of daughter cell assembly, is altered in PPKL-depleted parasites. Thus, we propose that PPKL regulates daughter parasite development by influencing the Crk1-dependent signaling pathway.

**IMPORTANCE:**

*Toxoplasma gondii* can cause severe disease in immunocompromised or immunosuppressed patients and during congenital infections. Treating toxoplasmosis presents enormous challenges since the parasite shares many biological processes with its mammalian hosts, which results in significant side effects with current therapies. Consequently, proteins that are essential and unique to the parasite represent favorable targets for drug development. Interestingly, *Toxoplasma*, like other members of the phylum Apicomplexa, has numerous plant-like proteins, many of which play crucial roles and do not have equivalents in the mammalian host. In this study, we found that the plant-like protein phosphatase PPKL appears to be a key regulator of daughter parasite development. With the depletion of PPKL, the parasite shows severe defects in forming daughter parasites. This study provides novel insights into the understanding of parasite division and offers a new potential target for the development of antiparasitic drugs.

## INTRODUCTION

Apicomplexa phylum species are parasites of humans and other animals, causing various diseases, such as malaria, toxoplasmosis, and cryptosporidiosis. Apicomplexa has highly specialized organelles, such as micronemes, rhoptries, and polar rings, which are critical in the propagation and virulence of these parasites (1). Among these unique organelles is the non-photosynthetic plastid known as the apicoplast, which is thought to have originated from an algal endosymbiont engulfed by a common ancestor of the current apicomplexans (2). This secondary endosymbiotic event is also thought to have resulted in significant horizontal gene transfer from the endosymbiont to the nuclear genome during evolution (2). As a result, apicomplexan genomes retain a multitude of plant-like genes. Many of these plant-like genes encode unique proteins essential for parasite biology, including the ApiAP2 transcription factors, which are key regulators of apicomplexan life cycle progression and differentiation (3), and the calcium-dependent protein kinases that regulate motility, invasion, and egress (4–7). As no homologs of most of these plant-like proteins are present in mammalian cells, they serve as favorable drug targets for the development of antiparasitic drugs.

The PPP family protein phosphatase PPKL, which contains a Kelch domain at its N-terminal region, is found in land plants and green alga and, interestingly, in alveolates, including apicomplexans (8). All available apicomplexan genomes encode a single PPKL (8). In contrast, *Arabidopsis* encodes four PPKLs, including brassinosteroid-insensitive 1 (BRI1) suppressor (BSU1) and BSU-LIKE 1, 2, and 3 (BSL1,2,3) (9). BSU1 is the most well-studied PPKL and is central to the brassinosteroid signaling pathway in *Arabidopsis* (10, 11). Brassinosteroids (BRs) are essential growth-promoting hormones in plants, which are ligands of BRI1, a receptor kinase located in the plasma membrane (12, 13). BRI1 works in conjunction with the co-receptor BRI1-associated kinase 1 (BAK1) (14). In the absence of BRs, BRI1 is inactive and bound to the inhibitor protein BRI1 kinase inhibitor 1 (BKI1) (15). When BR binds to the extracellular domains of BRI1 and BAK1, the cytoplasmic kinase domain of BRI1 is activated and phosphorylates BKI1, leading to its dissociation (16, 17). BRI1 is then fully activated by the formation of a heterodimeric complex with BAK1 and phosphorylation of the cytoplasmic domains of both kinases (16, 17). Activated BRI1 initiates a signaling cascade that leads to the activation of BSU1 by phosphorylation (18, 19). Activated BSU1 then dephosphorylates brassinosteroid-insensitive 2 (BIN2), a plant homolog of glycogen synthase kinase 3 (GSK3)-like serine/threonine kinase, which leads to its degradation (19, 20). In the absence of brassinosteroids, phosphorylated and active BIN2 phosphorylates two transcription factors, BZR1 (Brassinazole-resistant 1) and BES1 (BRI1-EMS suppressor 1), leading to their retention in the cytoplasm and proteasomal degradation mediated by ubiquitination (21–23). Thus, dephosphorylation of BIN2 by BSU1 leads to its degradation, which allows BZR1 and BES1 to act in the nucleus to activate the expression of BR-responsive genes (16, 24).

While apicomplexans do not produce BRs and lack the receptors and most of the proteins involved in brassinosteroid signaling, they express PPKLs with strong homology to BSU1 (25). To date, the study of PPKL in apicomplexans has been limited to *Plasmodium*, where it was found to be dominantly expressed in female gametocytes and ookinetes, and deletion of PfPPKL resulted in defects in the integrity of apical structures, motility, and mosquito invasion (25, 26). To further explore the function of PPKL in apicomplexans, we have focused on PPKL in *Toxoplasma gondii*, an obligate intracellular parasite in the phylum Apicomplexa. *Toxoplasma* infection is prevalent in humans worldwide. Although *Toxoplasma* infection poses a minimal danger to people with healthy immune systems, it constitutes a considerable threat to immunocompromised patients and during congenital infections (27, 28). The available drugs that can treat toxoplasmosis are very limited and have significant toxic side effects (29). Here, we show that PPKL has a highly dynamic localization pattern during parasite division and that, importantly, it is essential for parasite division. Moreover, we show that PPKL is an important part of the signaling pathways that control the cell cycle, division, and cytoskeletal regulation. Therefore, this work sheds light on the unique processes by which this important pathogen divides and reveals an essential enzyme that could serve as a target for much-needed therapeutics.

## RESULTS

### PPKL exhibits multiple cellular localizations and is associated with the progression of daughter parasite formation

**Fig 1:**
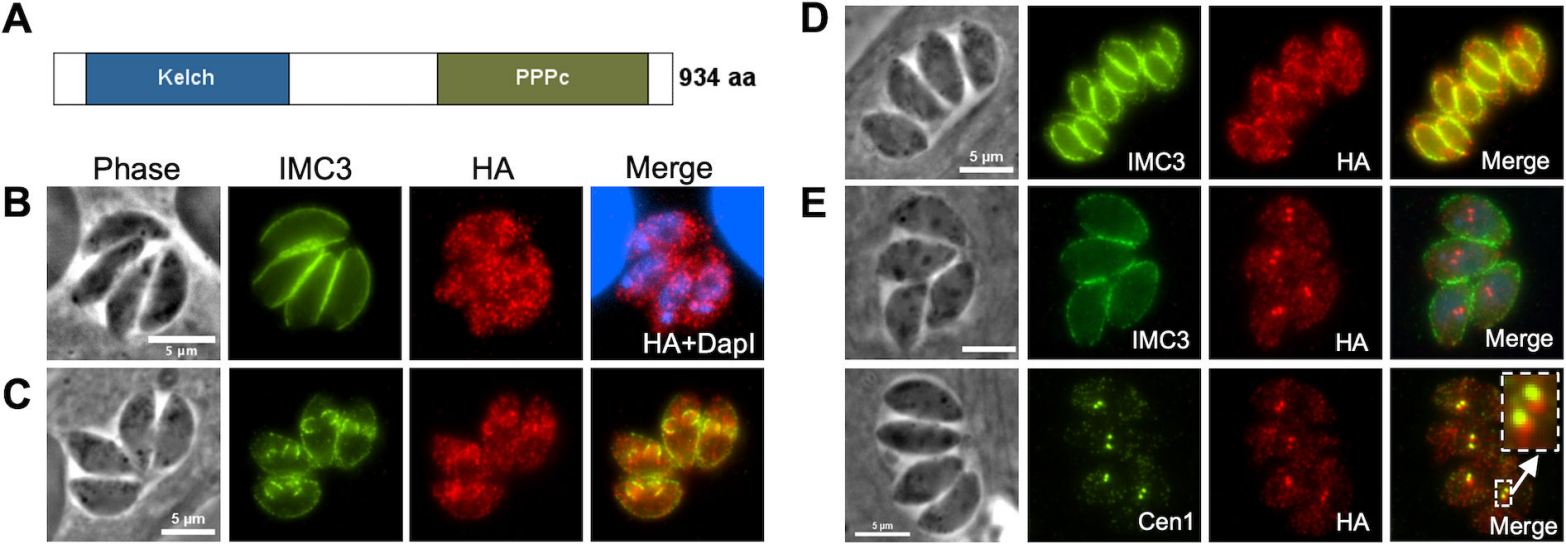
PPKL shows dynamic localization during division. (A) Schematic of PPKL in *Toxoplasma*. (B to E) Intracellular parasites of the PPKL^HA^ strain were stained with anti-HA antibodies to monitor PPKL localization in non-dividing parasites (**B**) and in parasites in the mid (**C**), late (**D**), or early (**E**) stages of division. Division was monitored with antibodies against IMC3 (**B to E**) or centrin 1 (**E**). The white arrow in panel E indicates the area expanded in the box. Scale bar: 5 µm.

PPKL (TGGT1_290170) in *Toxoplasma* consists of a sequence of 934 amino acids (aa), which exhibits a structural organization like its homolog in plants and other apicomplexans, featuring six Kelch motifs located in the N-terminal region, followed by the protein phosphatase domain situated in the C-terminal region (Fig. 1A). To determine where PPKL localizes within the parasite, we used a CRISPR/Cas9-based strategy to generate a strain in which the endogenous gene encoded a C-terminal triple hemagglutinin (3xHA) epitope tag. The resulting strain, Δ*ku80*:PPKL.3xHA (referred to as PPKL^HA^ hereafter), was used for immunofluorescence assays (IFAs) of intracellular parasites. As transcriptomic data show that *PPKL* expression varies during the cell cycle (ToxoDB), we co-stained parasites for the inner membrane complex (IMC) protein IMC3 to monitor parasite division and daughter cell formation. In non-dividing parasites, PPKL is present throughout the parasite and can be detected in both the cytoplasm and the nucleus (Fig. 1B). To confirm that PPKL is present in the nucleus, we performed cytoplasmic and nuclear fractionation of PPKL^HA^ parasites and then compared the ratio of PPKL in the nuclear fraction with that of the exclusively cytoplasmic protein eIF2α (30). The results showed that PPKL was significantly enriched in the nuclear fraction in relation to eIF2α (Fig. S1).

Interestingly, in dividing parasites, PPKL appears to be associated with the daughter parasites and is enriched in the cortical cytoskeleton of the daughter parasites (Fig. 1C and D). Intriguingly, we found that in a small fraction (6.5% ± 0.7%) of parasites, PPKL was heavily enriched in two distinct bright spots, which partially overlapped with the two duplicated centrosomes labeled by the anti-centrin 1 antibody (Fig. 1E). This localization suggests that PPKL may be present in the daughter parasite buds at a very early stage of division before the formation of the IMC of the daughter cells (Fig. 1E).

**Fig 2:**
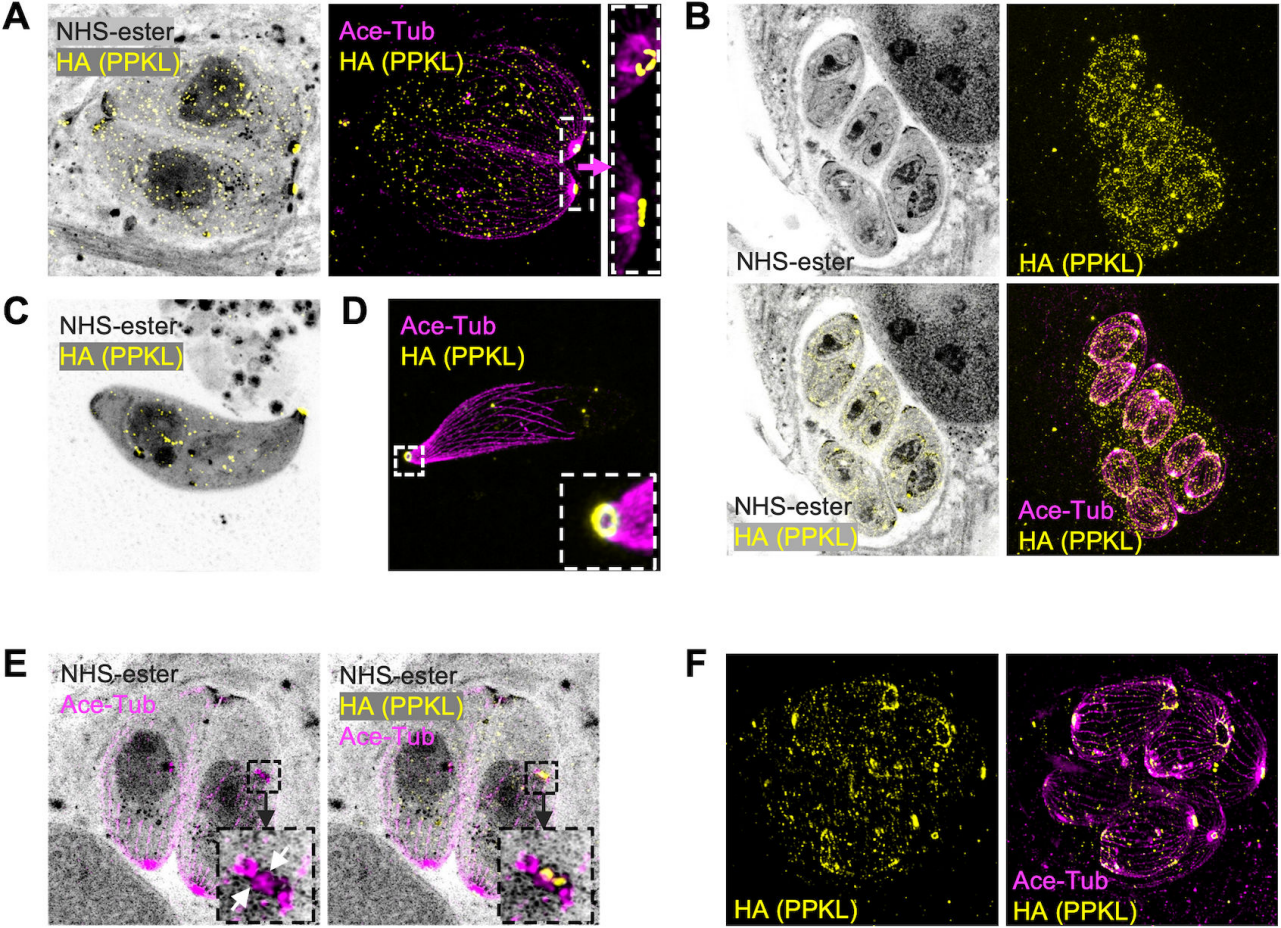
Ultrastructure expansion microscopy reveals PPKL in basal and apical structures. Intracellular and extracellular parasites were fixed with paraformaldehyde, expanded in acrylamide gels, and stained with NHS-ester, anti-HA, and anti-acetylated tubulin. Images were captured by LSM 900 with Airyscan. (A) Image of intracellular non-dividing parasites. The white box frames the region expanded to the right of the arrow. (B) Images of intracellular dividing parasites. (C and D) Images of extracellular parasites. The white framed zone in panel D is zoomed in and shown in the lower right corner. (E) Images of intracellular parasites. The parasite on the right has started daughter parasite assembly, as shown by duplicated centrosomes, preconoidal regions (yellow dots labeled by PPKL), and apical polar rings (indicated with white arrows), which are framed in a black box. The box is enlarged to the left showing the acetylated tubulin signal and to the right showing both anti-acetylated tubulin and anti-HA. (F) Images show intracellular parasites in a late stage of division.

For a more detailed analysis of PPKL localization, we performed ultrastructure expansion microscopy (U-ExM) (31, 32). To observe the protein and other parasite features of interest, we employed NHS-ester to stain all proteins, an acetylated tubulin antibody to visualize microtubules, an anti-HA antibody to detect HA-tagged PPKL, and Draq5 to label DNA. As we have seen by standard IFA, analysis of U-ExM images of parasites that have expanded in volume by approximately 100-fold corroborates that PPKL is present throughout the parasite in non-dividing ones and appears to be enriched in the cortical cytoskeleton of daughter parasites (Fig. 2A and B). Interestingly, U-ExM allowed us to observe that PPKL is enriched at the apical end of both mother and daughter parasites in a ring-like pattern (Fig. 2A and B). To more clearly determine whether the PPKL ring overlapped with the apical polar ring or the preconoidal region, we performed U-ExM for extracellular parasites to observe parasites with extended conoids. In extracellular parasites with protruded conoids, the PPKL ring was on the apical end of the conoid (Fig. 2C and D), suggesting that PPKL is present in or near the preconoidal region. Remarkably, we observed that during the earliest stages of division, PPKL is one of the earliest components present in the daughter parasite scaffold (Fig. 2E), which only contained two distinct regions, one labeled by PPKL and a second one, presumably the apical polar rings (indicated with white arrows), labeled by an anti-acetylated tubulin antibody. In addition, we observed that in daughter parasites late in the division, PPKL was enriched in the basal complex ring (Fig. 2F), suggesting that PPKL may be involved in terminating the extension of microtubules or in the contraction of the basal complex ring. In sum, both IFA and U-ExM showed that PPKL is present in multiple cellular locations and that the dynamic localization pattern is associated with the progression of daughter parasite formation, suggesting that PPKL may play multiple roles during daughter parasite development.

### Depletion of PPKL in parasites leads to the disruption of parasite division

**Fig 3:**
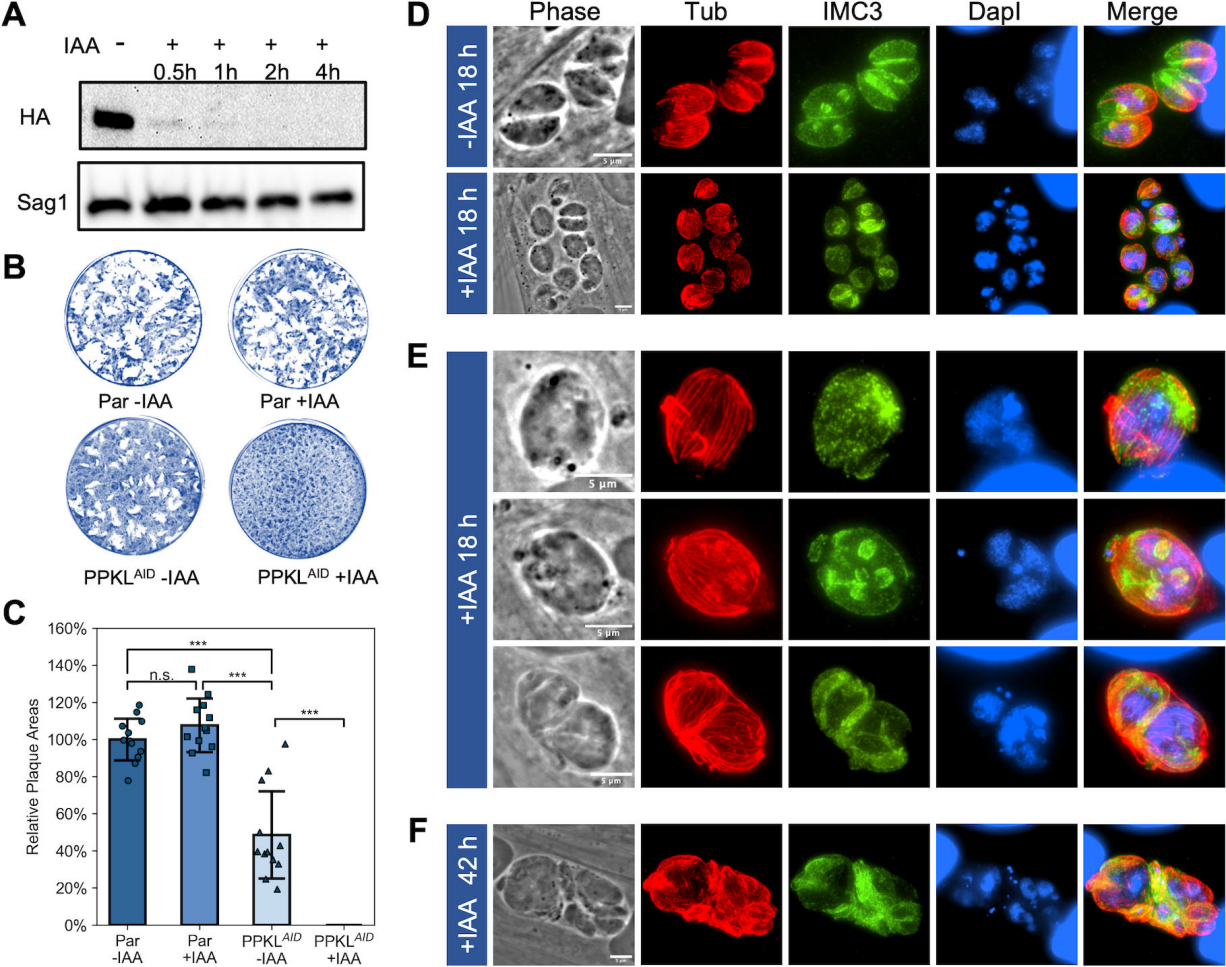
Depletion of PPKL leads to disruption of parasite division. (A) Shown is a representative Western blot of protein extract of the AID-tagged PPKL strain (PPKL^AID^) treated with auxin (IAA) for the times indicated and probed for the HA epitope tag. The protein Sag1 was used as a loading control. (B and C) Parasites from the parental (par) or PPKL^AID^ were grown for 6 days in the absence or presence of auxin (IAA) and allowed to form plaques. Panel B shows representative plaque assays. The graph in panel C is the average plaque area formed by the two strains, based on data collected from four biological replicates, with three experimental replicates for each. For each biological replicate, the data were normalized to the average plaque area of the parental strain without IAA treatment. The error bars represent standard deviations. ***P < 0.001. n.s., no significance (Student’s *t*-test, two tails, unequal variance). (D to F) PPKL^AID^ parasites were grown with/without auxin for 18 hours (**D and E**) or 42 hours (**F**) and analyzed by IFA. The cultures were stained with anti-*Toxoplasma* β-tubulin, anti-IMC3, and DapI. Scale bar: 5 µm.

Through a *Toxoplasma* genome-wide CRISPR screen, PPKL was assigned a fitness score of −5.02 (33), suggesting that it is essential for parasite survival and, therefore, that knockout of the *PPKL* gene is not likely possible. Accordingly, to investigate its function, we generated a conditional knockdown strain by using the auxin-induced degradation (AID) system (34). For this purpose, we inserted AID-3xHA prior to the stop codon of the endogenous *PPKL* gene using CRISPR-mediated gene editing. Western blot of the resulting PPKL^AID^ strain showed that treatment with auxin almost completely depleted PPKL within 1 hour (Fig. 3A). To determine if PPKL is required for *Toxoplasma* propagation, we performed plaque assays of both the parental and PPKL^AID^ strains with and without auxin. Consistent with the low fitness score, PPKL^AID^ parasites treated with auxin failed to form any plaques (Fig. 3B and C), indicating that PPKL is essential for parasite propagation. In addition, we observed that untreated PPKL^AID^ parasites formed fewer and smaller plaques than the parental strain (Fig. 3B and C), which may be due to the lower expression of PPKL-AID, as evident from the comparison with the PPKL^HA^ parasite (Fig. S2), or perhaps because the fusion of AID to PPKL may slightly impair its function.

To determine the cellular consequences of PPKL depletion, we performed IFA to observe the morphology of PPKL^AID^ parasites treated with auxin. Briefly, we added auxin to the culture 2 hours after infection and allowed parasites to grow for 18 hours in total. IFAs were performed using antibodies against *Toxoplasma* β-tubulin and IMC3 to monitor the formation of daughter cells. Strikingly, in the auxin-treated PPKL^AID^ cultures, most vacuoles contained only one extremely swollen parasite, whereas most untreated vacuoles had undergone two division cycles with an average division count of 1.64 ± 0.06 (Fig. 3D). Interestingly, the swollen parasites in the auxin-treated cultures contained a large amount of DNA, far exceeding the amount of a normal single parasite (Fig. 3D and E). However, most (71% ± 6%) of these swollen parasites did not have any daughter parasites (Fig. 3E, top row), and only a few (10% ± 2%) contained a varied number of seemingly abnormal daughter parasites (Fig. 3E, middle row). Moreover, there were some (18% ± 4%) vacuoles containing two expanded parasites, similar to an increased amount of DNA (Fig. 3E, bottom row). When we extended the treatment of auxin to 2 days (~42 hours), the vacuoles became more disorganized and had more abnormal parasites, and swollen parasites had a further accumulation of DNA (Fig. 3F), implying that division was progressing, albeit abnormally. In summary, PPKL-depleted parasites have deficiencies in daughter parasite formation. Still, their ability to replicate DNA appears relatively unimpaired, resulting in division uncoupling.

### Depletion of PPKL does not affect the replication of centrosomes

**Fig 4:**
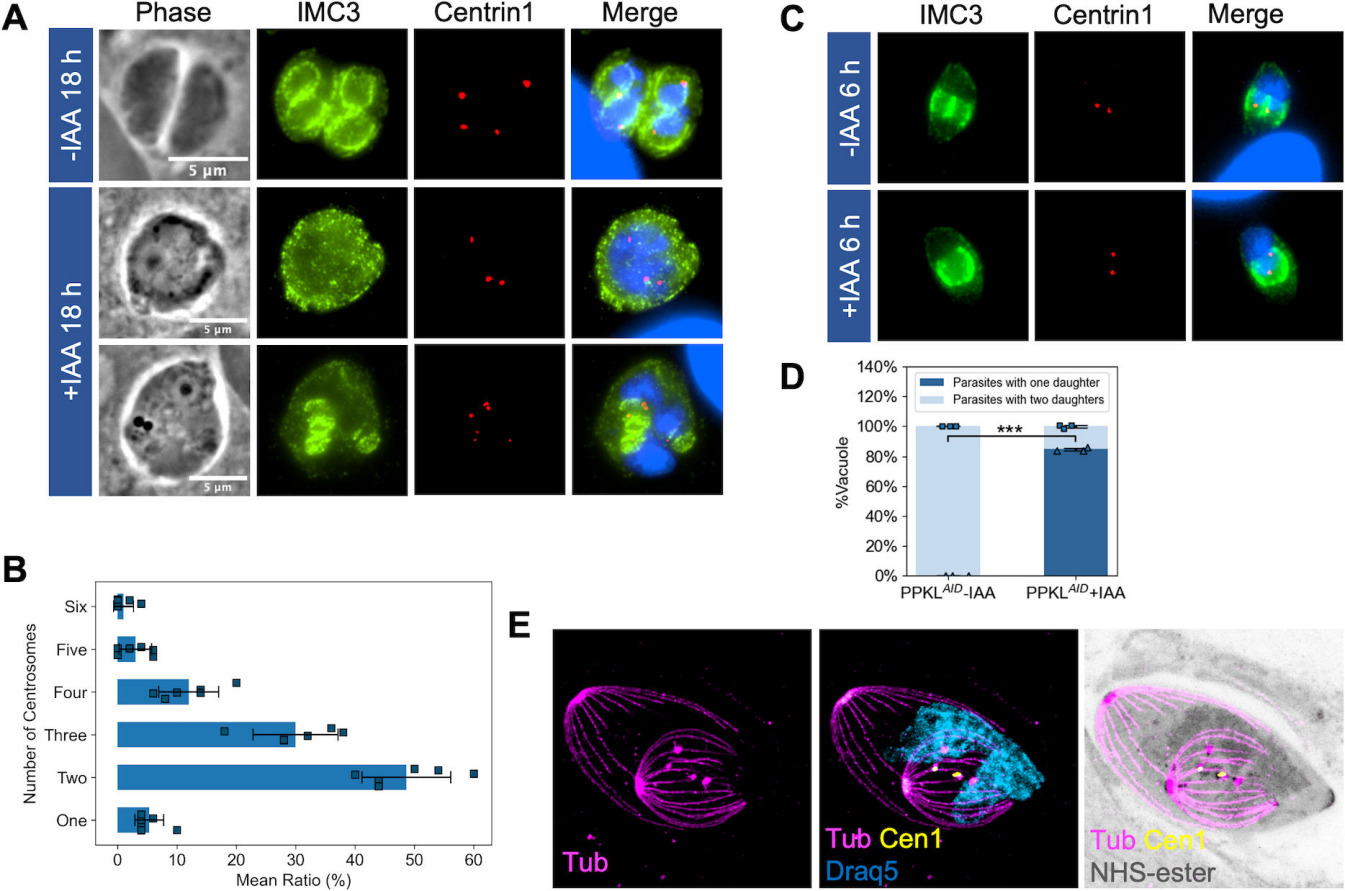
Depletion of PPKL does not affect the replication of centrosomes. Centrosome duplication of PPKL^AID^ grown with and without auxin was monitored by IFA using antibodies against centrin 1. (A) IFA of parasites grown with/without IAA for 18 hours and stained with anti-IMC3, anti-centrin 1 antibodies, and DapI. (B) Quantification (mean ± SD) of the number of centrosomes of single swollen PPKL^AID^ parasites treated with IAA for 18 hours. (C) IFA of PPKL^AID^ parasites grown with/without IAA for 6 hours. (D) Quantification (mean ± SD) of dividing PPKL^AID^ parasites treated with/without IAA for 6 hours with one or two daughters. Three biological replicates were performed, with 50 parasites counted for each replicate. ***P < 0.001 (Student’s *t*-test, two tails, unequal variance). (E) U-ExM of one daughter containing PPKL^AID^ parasites treated with IAA for 6 hours and stained with anti-acetylated tubulin, anti-centrin 1, NHS-ester, and Draq5. Scale bar: 5 µm.

During endodyogeny, the two-daughter parasite scaffolds are assembled near the two centrosomes in a one-to-one correspondence (35). Centrosome duplication has been shown to occur before the formation of daughter parasites, and it is needed for the division to initiate (36, 37). Based on the significant defects in parasite division in the PPKL mutant, we monitored centrosome duplication upon PPKL depletion. As expected, untreated dividing parasites had exactly two centrosomes in each mother cell (Fig. 4A). Similarly, those swollen parasites treated with auxin and cultured for 18 hours predominantly had at least two centrosomes (Fig. 4A and B), regardless of whether they contained daughter parasites or not, implying that centrosome duplication was still taking place. Interestingly, we often observe the swollen parasites in the auxin-treated cultures that have more than two centrosomes (Fig. 4A and B). It should be considered that by the equivalent amount of time in culture, the untreated parasites have undergone two division cycles (division count: 1.64 ± 0.06). Thus, the presence of more than two centrosomes within one parasite is consistent with our observation that PPKL depletion leads to division (i.e., centrosome and DNA duplication) and daughter parasite formation being uncoupled.

To further examine centrosome duplication in PPKL-depleted parasites, we added auxin right after infection and allowed the parasites to grow for 6 hours, which allowed us to monitor the first division cycle of the parasites after PPKL depletion. IFA showed that few parasites had detectable daughter parasites for both the control and auxin-treated parasites. Interestingly, among the parasites that were dividing, 85% ± 1.15% of those from auxin-treated parasites had only one daughter parasite (Fig. 4C and D), and 95% ± 1.15% of the parasites containing only one daughter had two centrosomes. We also observed that in the treated parasites, the single-daughter parasite appeared to be morphologically unusual and larger than normal. We explored this phenotype using U-ExM and observed that the microtubule system of the single-daughter parasite appeared to be normal, except that the gaps between the cortical microtubules were wider, which correlates with the larger appearance of the parasites (Fig. 4E). As shown in Fig. 4E, we also observed parasites in which there were two centrosomes. Taken together, our results suggest that although division is impaired upon depletion of PPKL, centrosome duplication remains unaffected.

### Depletion of PPKL reduces the stability of microtubules

**Fig 5:**
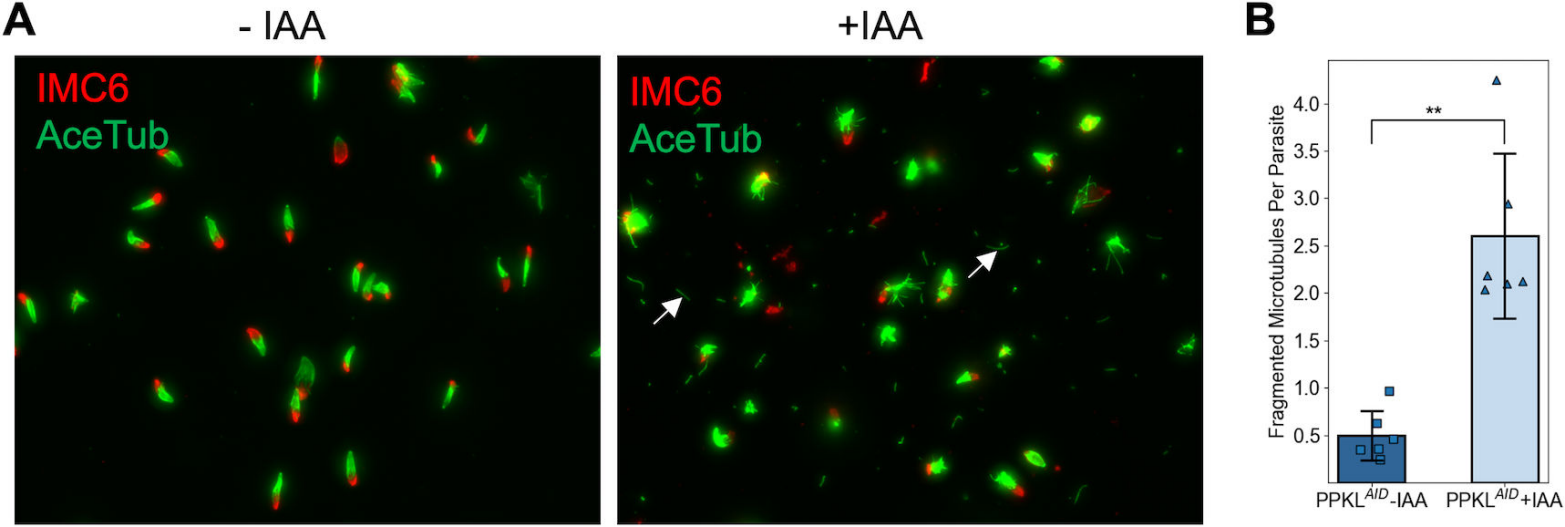
Depletion of PPKL reduces the stability of microtubules. The cortical cytoskeleton was extracted from PPKL^AID^ parasites treated with IAA or ethanol for 6 hours. (A) IFA of the extracted cortical cytoskeleton used anti-acetylated tubulin and anti-IMC6 antibodies to monitor the microtubules and IMC, respectively. Examples of fragmented microtubules are indicated by white arrows. (B) Quantification of fragmented microtubules normalized to the number of cortical cytoskeletons. The bars indicate the average number of fragmented microtubules from each cortical cytoskeleton. The error bars represent standard deviations. Three biological replicates, each consisting of two experimental replicates, were performed. For each experimental replicate, 10 random fields of view were selected to count the number of cortical cytoskeleton and fragmented microtubules. **P < 0.01 (Student’s *t*-test, two tails, unequal variance).

A previous study has shown that the knockout of the PPKL gene in *Plasmodium* led to apical microtubule disorganization and dissociation from the IMC (25). Interestingly, PPKL also appears to be required to maintain the order and/or stability of microtubules in *Toxoplasma,* as observed through the disruption of the compact structure of the cortical cytoskeleton in the knockdown strain. To confirm this observation, we treated PPKL^AID^ parasites that had undergone two to three rounds of division with auxin for 6 hours and isolated their cortical cytoskeleton using sodium deoxycholate. As expected, the cortical cytoskeleton from the control parasites appeared more compact with few fragmented microtubules (Fig. 5A). In contrast, the cortical cytoskeleton from auxin-treated parasites appeared more disordered with five times more fragmented microtubules than the control (Fig. 5A and B), suggesting that PPKL is involved in the regulation of the stability and compact structure of the cortical microtubule system.

### IMC29 and DYRK1 are likely PPKL functional partners

To determine the molecular mechanisms responsible for PPKL knockdown phenotypes, we set out to identify functional partners and putative substrates of PPKL. We first performed standard co-immunoprecipitation (Co-IP) using anti-HA-conjugated magnetic beads to precipitate HA-tagged PPKL from the PPKL^HA^ parasites. However, this approach failed to identify PPKL interactors, suggesting that either PPKL works on its own or it interacts with other proteins and substrates transiently or with low affinity. Accordingly, we used disuccinimidyl sulfoxide (DSSO) to crosslink the protein samples before performing Co-IP. The results of the tandem mass spectrometry (MS/MS) analysis indicated that two proteins significantly co-precipitated with PPKL: IMC29 (TGGT1_243200) and DYRK1 (TGGT1_204280) (Supplemental Data Set S1). The inner membrane complex protein IMC29 has been identified as a crucial component of early daughter buds with a substantial contribution to parasite division (27). DYRK1 is a cell cycle-related protein kinase of unknown function.

As a complementary approach to Co-IP, we used the TurboID proximity labeling method (38), which can identify neighboring and interacting proteins, including substrates. To this end, we endogenously fused the engineered biotin ligase TurboID and a 3xHA epitope tag to the C-terminus of PPKL. IFA of the obtained parasite strain showed that PPKL^TurboID.HA^ has the same localization pattern as PPKL^HA^ (Fig. S3A). We then confirmed that the TurboID portion of the fusion protein was active by incubating PPKL^TurboID.HA^ parasites with D-biotin and performed Western blot, which showed a significant increase in biotinylated proteins compared to the same parasite strain without biotin treatment (Fig. S3B). To identify interacting and neighboring proteins, we incubated PPKL^TurboID.HA^ or parental strain parasites with D-biotin and isolated biotinylated proteins for MS/MS for two experimental replicates. We applied the following criteria to the resulting list of putative interactors: in combination with two replicates, (i) they are identified in both replicates; and (ii) the *P* value of Fisher’s exact test for the combined data is <0.05. In this manner, we obtained a list of 97 putative PPKL neighboring and/or interacting proteins (Table 1; Supplemental Data Set S2). To further analyze this list of proteins, we investigated their potential molecular functions based on functional annotations in ToxoDB. Out of the 97 proteins, 19 are related to the cortical cytoskeleton system, including the IMC, apical complex, basal complex, and microtubules; seven are potentially related to vesicle transport; five are potentially related to RNA splicing (Table 1). Moreover, there are three protein kinases (TGGT1_231070, DYRK1, and SRPK1) and one protein phosphatase, PPM2A. Notably, both IMC29 and DYRK1, which were identified as putative interactors via crosslinking and Co-IP, were identified with the TurboID approach, suggesting that they are highly likely functional partners of PPKL.

**TABLE 1:** Putative PPKL neighboring proteins^*a*^

Gene ID	Annotation	Fold change	*P* value	Peptides		Comments
				Control	PPKL-TurboID	
TGGT1_244380	Cactin	INF	1.09E-18	0	363	c
TGGT1_293180	NADP-specific glutamate dehydrogenase	INF	3.58E-14	0	169	
TGGT1_290170	PPKL	INF	4.18E-14	0	167	
TGGT1_245560	Hypothetical protein	INF	1.54E-13	0	151	
TGGT1_232340	PPM2A	INF	4.30E-12	0	116	
TGGT1_313270	Hypothetical protein	INF	1.84E-11	0	103	
TGGT1_269410	Hypothetical protein	INF	2.07E-11	0	102	
TGGT1_311230	BCC7	63.00	1.36E-10	2	126	a
TGGT1_310220	Hypothetical protein	INF	1.56E-10	0	86	
TGGT1_203780	Hypothetical protein	INF	1.56E-10	0	86	
TGGT1_321620	DRPB	INF	2.06E-10	0	84	b
TGGT1_243200	IMC29	INF	3.60E-10	0	80	a
TGGT1_291180	Hypothetical protein	INF	1.62E-09	0	70	
TGGT1_227800	EPS15	INF	2.23E-09	0	68	b
TGGT1_244120	Hypothetical protein	INF	1.24E-08	0	58	
TGGT1_220270	IMC6	INF	1.48E-08	0	57	a
TGGT1_214180	EpsL	INF	2.15E-08	0	55	b
TGGT1_259640	Nucleoporin autopeptidase	INF	2.60E-08	0	54	
TGGT1_294360	Putative ubiquitin-specific protease 39 isoform 2	INF	4.65E-08	0	51	c
TGGT1_268950	Hypothetical protein	INF	6.95E-08	0	49	
TGGT1_275490	Hypothetical protein	INF	1.30E-07	0	46	
TGGT1_311400	SEC31A	INF	1.99E-07	0	44	b
TGGT1_212140	Hypothetical protein	INF	4.90E-07	0	40	
TGGT1_253440	SRPK1	50.00	5.70E-07	1	50	c
TGGT1_292950	AC10	INF	1.28E-06	0	36	a
TGGT1_262150	K13	INF	2.73E-06	0	33	b
TGGT1_231640	IMC1	8.54	2.94E-06	24	205	a
TGGT1_231070	Protein kinase	INF	9.12E-06	0	29	a
TGGT1_221660	DEAD/DEAH box helicase domain-containing protein	INF	9.12E-06	0	29	c
TGGT1_204280	DYRK1	INF	9.12E-06	0	29	
TGGT1_207370	Hypothetical protein	INF	1.13E-05	0	28	
TGGT1_313790	Hypothetical protein	INF	1.13E-05	0	28	
TGGT1_230210	IMC10	INF	1.46E-05	0	27	a
TGGT1_201700	SEC13	34.00	2.18E-05	1	34	b
TGGT1_275350	TBC domain-containing protein	INF	3.23E-05	0	25	b
TGGT1_265870A	Pantoate-beta-alanine ligase	INF	3.23E-05	0	25	
TGGT1_320080	Hypothetical protein	INF	3.87E-05	0	24	
TGGT1_298610	GYF domain-containing protein	INF	3.87E-05	0	24	
TGGT1_235340	ISC1	INF	4.98E-05	0	23	a
TGGT1_250700	Hypothetical protein	INF	9.51E-05	0	22	
TGGT1_216000	IMC3	13.00	1.02E-04	3	39	a
TGGT1_228150	Hypothetical protein	INF	1.07E-04	0	21	
TGGT1_214880	AC4	INF	1.35E-04	0	20	a
TGGT1_260580	Hypothetical protein	INF	1.35E-04	0	20	
TGGT1_260600	TgPuf1	INF	1.35E-04	0	20	
TGGT1_223420	DnaJ domain-containing protein	INF	1.35E-04	0	20	
TGGT1_285500	Hypothetical protein	INF	1.35E-04	0	20	
TGGT1_237290	Hypothetical protein	INF	1.35E-04	0	20	
TGGT1_263520	SPM1	INF	1.35E-04	0	20	a
TGGT1_231630	IMC4	5.89	1.98E-04	18	106	a
TGGT1_205380	Fructose bisphosphatase I	INF	2.60E-04	0	19	
TGGT1_213392	Surface antigen repeat-containing protein	INF	2.60E-04	0	19	
TGGT1_296010	Phosphatidylinositol 3- and 4-kinase	INF	2.60E-04	0	19	
TGGT1_305340	Corepressor complex CRC230	INF	2.94E-04	0	18	
TGGT1_310440	MORN1	INF	2.94E-04	0	18	a
TGGT1_291950	RNA recognition motif-containing protein	INF	2.94E-04	0	18	
TGGT1_294730	Hypothetical protein	INF	2.94E-04	0	18	
TGGT1_230940	Hypothetical protein	6.45	3.25E-04	11	71	
TGGT1_294610	SET	INF	6.62E-04	0	17	
TGGT1_258540	Phosphoglycerate mutase family protein	INF	6.62E-04	0	17	
TGGT1_313430	Hypothetical protein	INF	6.62E-04	0	17	
TGGT1_219710	Hypothetical protein	INF	6.70E-04	0	16	
TGGT1_280370	Hypothetical protein	INF	8.21E-04	0	15	
TGGT1_244470	RNG2	INF	8.21E-04	0	15	a
TGGT1_260540	IMC14	INF	1.53E-03	0	14	a
TGGT1_273560	Kinesin B	INF	1.53E-03	0	14	a
TGGT1_253430	Putative asparagine synthetase	6.50	1.69E-03	6	39	
TGGT1_282070	Hypothetical protein	INF	1.82E-03	0	13	
TGGT1_249440	Hypothetical protein	INF	1.82E-03	0	13	
TGGT1_214970	Putative DNA replication licensing factor	INF	3.39E-03	0	12	
TGGT1_286580	IMC17	INF	3.39E-03	0	12	a
TGGT1_310950	AP2XI-3	INF	3.39E-03	0	12	
TGGT1_306660	RNA pseudouridine synthase superfamily protein	INF	3.39E-03	0	12	
TGGT1_270770	PWI domain-containing protein	INF	3.39E-03	0	12	
TGGT1_218960	AP2XII-1	INF	3.39E-03	0	12	
TGGT1_248700	IMC12	16.00	3.87E-03	1	16	a
TGGT1_224850	Putative polyadenylate-binding protein	10.00	6.24E-03	2	20	
TGGT1_201680	Putative eukaryotic initiation factor-3 subunit 10	10.00	6.24E-03	2	20	
TGGT1_212260	Autoantigen p27	INF	7.36E-03	0	10	
TGGT1_270890	Hypothetical protein	INF	7.36E-03	0	10	
TGGT1_313910	RNA recognition motif 2 protein	INF	7.36E-03	0	10	
TGGT1_204160	GYF domain-containing protein	INF	7.74E-03	0	11	
TGGT1_313380	ILP1	14.00	8.05E-03	1	14	a
TGGT1_313010	DDX6	5.75	0.012	4	23	c
TGGT1_291140	CCR4-Not complex component, Not1 protein	INF	0.015	0	9	
TGGT1_315700	Hypothetical protein	INF	0.015	0	9	
TGGT1_313370	Hypothetical protein	INF	0.015	0	9	
TGGT1_309140	Transducin beta-like protein TBL1	INF	0.015	0	9	
TGGT1_207060	Ribonucleoside-diphosphate reductase small subunit	INF	0.015	0	9	
TGGT1_306220	Hypothetical protein	INF	0.015	0	9	
TGGT1_262950	Hypothetical protein	INF	0.015	0	9	
TGGT1_268630	YagE family protein	INF	0.016	0	8	
TGGT1_276890	Hypothetical protein	INF	0.016	0	8	
TGGT1_250820	Hypothetical protein	3.53	0.019	15	53	
TGGT1_269290	Hypothetical protein	INF	0.031	0	7	
TGGT1_258970	Hypothetical protein	INF	0.031	0	7	
TGGT1_246950	Hypothetical protein	INF	0.031	0	7	
TGGT1_273760	HSP70	2.48	0.043	71	176	

^*a*^ Listed are proteins identified by TurboID that met the following criteria: (i) identified in both replicates and (ii) *P* value of Fisher’s exact test for the combined data is < 0.05. Proteins related to the IMC, apical complex, basal complex, and microtubules are commented as “a”; proteins related to vesicle transport are commented as “b”; and proteins related to RNA splicing are commented as “c.” The number of peptides listed is the total between the two replicates.

### DYRK1 plays an important role in *Toxoplasma* division

**Fig 6:**
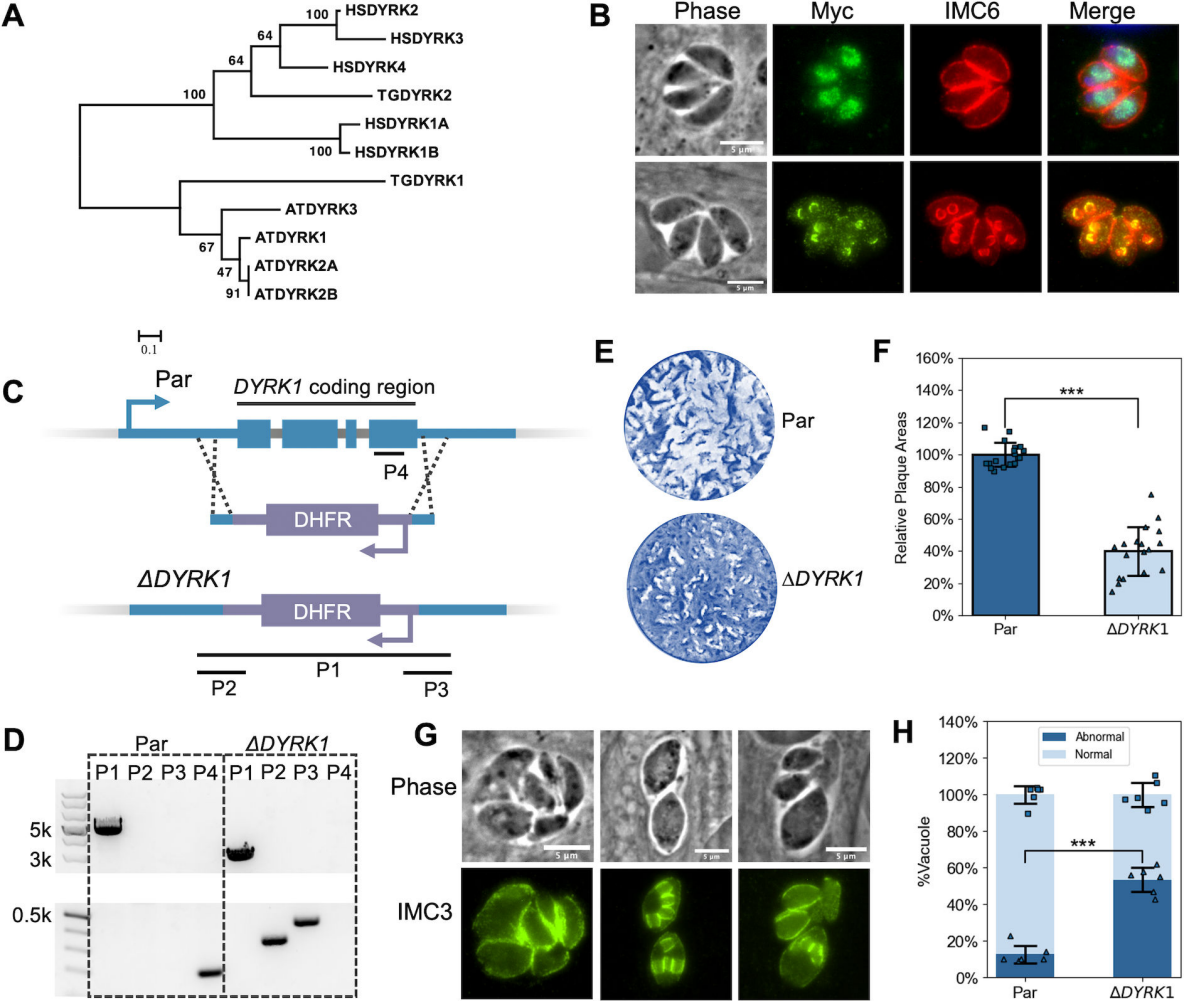
DYRK1 is a plant-like kinase and plays an important role in parasite division. (A) Phylogenetic analysis of DYRK sequences from humans (HSDYRK1A, 1B, 2, 3, and 4), *Arabidopsis* (ATDYRK1, 2A, 2B, and 3), and *Toxoplasma* (TGDYRK1 and 2). Alignment and tree construction details and accession numbers are listed in Materials and Methods. (B) IFA of parasites expressing Myc-tagged DYRK1 using anti-Myc and anti-IMC6 antibodies. The upper panel shows non-dividing parasites, while the lower panel shows dividing ones. Scale bar: 5 µm. (C) The diagram depicts the CRISPR/Cas9-mediated strategy used to disrupt the *DYRK1* gene. P1, 2, 3, and 4 are the four amplicons used to confirm the integration of the DHFR cassette between the Cas9 cutting sites and the deletion of the *DYRK1* gene. (D) Agarose gel of PCR products amplified from the genomic DNA extracted from Δ*DYRK1* parasites using the four sets of primers indicated in panel C. The P1 amplicon has a length of 2,928 bp in the Δ*DYRK1* genome and 4,787 bp in the parental genome. The P2, P3, and P4 amplicons have a length of 339 bp, 431 bp, and 196 bp, respectively. (E and F) Δ*DYRK1* and parental strain parasites were allowed to form plaques in culture for 6 days. A representative plaque assay (**E**) and the quantification are shown (**F**). The bars represent the relative average plaque areas, and the error bars represent standard deviations. Three biological replicates were performed, and six experimental replicates were included each time. For each biological replicate, the data were normalized to the average plaque area of the parental strain. (G) IFA images of Δ*DYRK1* parasites that show abnormal division. (H) Quantification (mean ± SD) of the ratio of vacuoles containing parasites displaying abnormal division or morphologies. ***P < 0.001 (Student’s *t*-test, two tails, unequal variance).

Based on the division phenotype of PPKL-depleted parasites, it is likely that PPKL participates in a signaling pathway that regulates parasite division. As signaling pathways often involve a chain of kinases and phosphatases, we focused on characterizing the function of DYRK1, which appears to be a putative PPKL interactor. Dual-specificity tyrosine-regulated kinase (DYRK) is a member of the CMGC group of kinases (39), a large and conserved family of kinases that play key roles in cell cycle regulation and many important signaling pathways (40). *Toxoplasma* encodes two DYRKs in its genome, and phylogenetic analysis (Fig. 6A), including human and *Arabidopsis* homologs, showed that DYRK1 was exclusively clustered with plant homologs, while DYRK2 (TGGT1_283480) was clustered with human homologs. Thus, both PPKL and DYRK1 are closer in homology to proteins from plants. To identify the localization of DYRK1, we endogenously fused a 3xMyc ectopic tag at its C-terminus with CRISPR/Cas9-mediated gene editing. IFA showed that in non-dividing parasites, DYRK1 localizes to the nucleus (Fig. 6B, top row), while in parasites undergoing division, it exclusively localizes to the IMCs of daughter parasite buds (Fig. 6B, bottom row). The dynamic localization of DYRK1 suggests that this kinase might have multiple roles throughout the division cycle of the parasite.

As a potential functional partner of PPKL, we sought to investigate whether the absence of DYRK1 manifests phenotypic similarities to PPKL knockdown. For this purpose, the *DYRK1* gene was disrupted through the replacement of the coding region with a DHFR expression cassette in the parental Δ*ku80* parasites by using the CRISPR/Cas9 system (Fig. 6C). The disruption of the *DYRK1* locus in the resulting Δ*DYRK1* strain was confirmed by PCR with four sets of primers (Fig. 6D). Plaque assays showed that Δ*DYRK1* parasites are less efficient at forming plaques as compared to the parental strain (40% ± 15% plaque efficiency relative to the parental strain; Fig. 6E and F). While these data suggest that DYRK1 is not essential, it appears to play an important role in parasite propagation. Importantly, IFA revealed that 53.5% ± 6.5% of vacuoles formed by Δ*DYRK1* parasites displayed abnormal parasite division (Fig. 6G and H), characterized by predominantly unsynchronized division, resulting in disorganized parasites within the vacuole and even atypical morphologies for some parasites, such as swollen parasites similar to what is observed upon PPKL depletion. The observed division defect, stemming from the deletion of DYRK1, strongly indicates the significant involvement of DYRK1 in regulating parasite division and a potential functional relationship between PPKL and DYRK1.

### PPKL influences the phosphorylation state of regulators of microtubule stability and cell division

**Fig 7:**
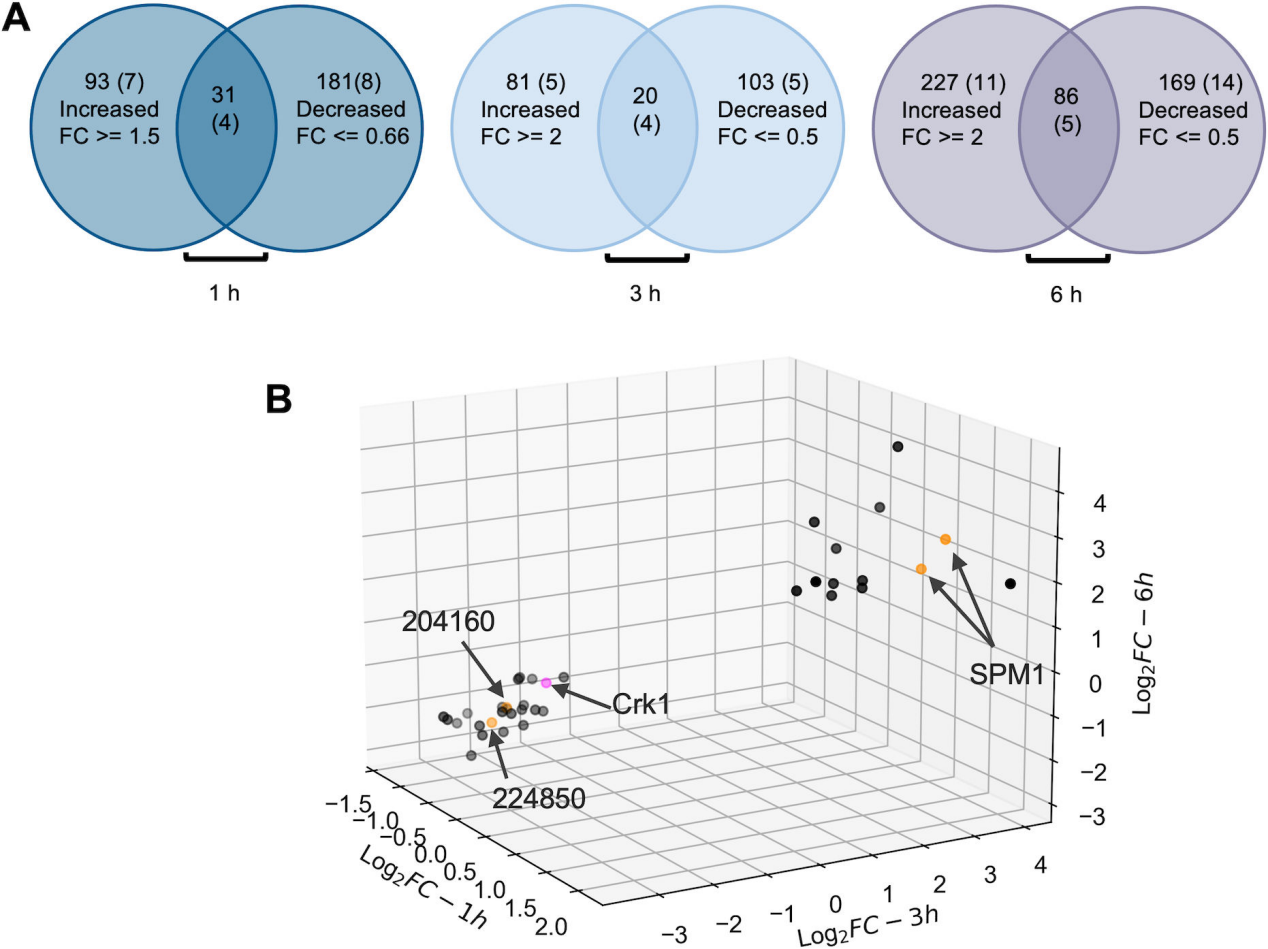
Phosphoproteomics analysis showed that depletion of PPKL results in increased phosphorylation of SPM1 and decreased phosphorylation of Crk1. (A) Venn diagrams showing the number of proteins with phosphopeptides that were more or less abundant in PPKL^AID^ parasites treated with auxin for 1, 3, and 6 hours. The overlap indicates the number of proteins that had phosphopeptides that increased and decreased in abundance upon PPKL depletion. The number of proteins that were also identified as PPKL neighboring proteins in each category is shown in parentheses. The fold change cutoffs are as follows: for the 1-hour timepoint sample set, phosphopeptides with FC >= 1.5 and FC <= 0.66; for the 3- and 6-hour timepoint sample sets, phosphopeptides with FC >= 2 and FC <= 0.5. (B) 3D scatter plot shows 35 phosphopeptides that are shared by all three timepoint sample sets after filtering with the cutoffs described above. Each dot in the 3D scatter plot represents one phosphopeptide. The dots shown in orange are peptides from PPKL neighboring proteins identified by TurboID analysis. The dot shown in pink represents the peptide from Crk1. *X*-axis: the log2 fold change of 1-hour samples. *Y*-axis: the log2 fold change of 3-hour samples. *Z*-axis: the log2 fold change of 6-hour samples.

To further explore the molecular mechanisms leading to the division phenotypes of PPKL-depleted parasites, we used tandem mass tag (TMT) quantitative mass spectrometry to compare the phosphoproteomes of PPKL^AID^ parasites treated with/without auxin. Briefly, PPKL^AID^ parasites were allowed to grow for 18 hours before adding either auxin or ethanol (vehicle control). After 1, 3, and 6 hours of treatment, cultures were harvested, and the parasites were released by syringe lysis, and samples were prepared for quantitative mass spectrometry. At the 6-hour timepoint, we identified 486 phosphopeptides from 313 proteins that were more than twofold abundant in the auxin-treated parasites (Fig. 7A; Supplemental Data Set S3). We also identified 425 phosphopeptides from 255 proteins that were more than twofold less abundant upon PPKL depletion (Fig. 7A; Supplemental Data Set S3). Interestingly, 86 proteins had both over- and under-phosphorylated peptides. Thus, we identified a total of 482 proteins whose phosphorylation state was PPKL dependent. Interestingly, 30 of the 97 proteins identified in the TurboID assay were among these 482 proteins (Supplemental Data Set S3), strongly validating the reliability of the phosphoproteome and interactome data. However, DYRK1 was not among these 30 proteins, and it had a 1.22-fold increase in phosphorylation of one residue and a 1.16-fold decrease in phosphorylation of another upon depletion of PPKL.

As expected, the results from the parasites treated for either 1 or 3 hours revealed a more limited number of peptides exhibiting PPKL-dependent phosphorylation status, especially for the 1-hour treated samples (Supplemental Data Set S4). Therefore, when filtering the quantitative proteomic data, we maintained a twofold change as the cutoff for 3-hour timepoint samples but reduced the fold change from 2 to 1.5 for the 1-hour timepoint samples to prevent missing important information. Thus, we identified 204 proteins (over: 124 peptides from 101 proteins; under: 153 peptides from 123 proteins; both: 20 proteins) that exhibited a PPKL-dependent phosphorylation state in the 3-hour timepoint samples and 304 proteins (over: 142 peptides from 124 proteins; under: 278 peptides from 212 proteins; both: 31 proteins) in the 1-hour timepoint samples (Fig. 7A; Supplemental Data Set S4). When combining the data to identify phosphopeptides shared by all three timepoint samples, we found that 13 phosphopeptides from 11 proteins were over-phosphorylated, and 22 phosphopeptides from 19 proteins were under-phosphorylated (Table 2; Fig. 7B). One of these proteins, TGGT1_214270, had both over- and under-phosphorylated peptides identified. Thus, we obtained a list of 29 proteins whose phosphorylation was affected by the lack of PPKL at all three timepoints of auxin treatment (Table 2).

**TABLE 2:** PPKL-dependent phosphopeptides^*a*^

Gene ID	Annotation	Peptides	Modification	Fold change (+IAA/−IAA)		
				1 hour	3 hours	6 hours
TGGT1_250830	SAC3/GANP family protein	[R].ESSKDLHAEK.[T]	1xPhospho [S]	4.37	15.87	3.737
TGGT1_263520	Microtubule-associated protein SPM1	[K].KLPSEEGSDYGYPQKPQK.[Y]	2xPhospho [S4(100); S8(100)]	2.12	12.83	3.099
		[K].KLPSEEGSDYGYPQKPQK.[Y]	1xPhospho [S4(100)]	2.43	14.78	5.256
TGGT1_235470	Myosin A	[R].SSDVHAVDHSGNVYK.[G]	1xPhospho [S/Y]	1.62	10.29	7.252
TGGT1_321680	Hypothetical protein	[K].EGREEEEEETASEEEDEHAEPK.[K]	2xPhospho [T10(100); S12(100)]	2.02	9.7	21.505
TGGT1_278660	Putative P-type ATPase4	[R].RFSSKRESTVGGSGTGHSQLGK.[S]	3xPhospho [S3(100); S4(100); S8(98.7)]	1.57	5.89	4.058
TGGT1_214270	Putative translation initiation factor IF-2	[K].DDSDDETKPAPPAK.[K]	1xPhospho [S/T]	1.98	2.53	2.772
		[R].RGGLSSDEEFEAK.[K]	2xPhospho [S5(100); S6(100)]	2.07	5.87	2.904
TGGT1_265250	RNA recognition motif-containing protein	[K].SKSPDFSELRK.[E]	2xPhospho [S1(100); S3(100)]	2.11	5.7	2.629
TGGT1_209600	Hypothetical protein	[K].EAQESSDDEDEDDAHFDGEDLK.[V]	1xPhospho [S]	1.87	4.51	2.718
TGGT1_289650	PEP carboxykinase I	[R].TQSSGSLKDSISFLEMLK.[K]	2xPhospho [T/S]	1.86	4.44	2.247
TGGT1_269180	MIF4G domain-containing protein	[R].RRGSNAGAAALPAGDGK.[V]	1xPhospho [S4(100)]	1.71	3.88	6.936
TGGT1_248420	Hypothetical protein	[R].SSSVSTIFK.[M]	2xPhospho [S2(99.5); S3(100)]	2.58	2.32	3.833
TGGT1_291930	RNA recognition motif-containing protein	[K].DVMMEEESDEDSDDEEKSERPVK.[K]	2xPhospho [S12(100); S18(98.8)]	0.49	0.25	0.257
		[K].NEKDVMMEEESDEDSDDEEK.[S]	2xPhospho [S11(100); S15(100)]	0.61	0.09	0.312
TGGT1_288380	Heat shock protein HSP90	[K].SVDKEITESEDEEKPAEDAEEK.[K]	2xPhospho [T7(100); S9(100)]	0.58	0.1	0.282
TGGT1_286790	Nuclear factor NF2	[K].KGLSADSDDASDKVSETKS.[-]	2xPhospho [S11(100); S15(98)]	0.42	0.16	0.193
		[K].KGLSADSDDASDKVSETK.[S]	2xPhospho [S7(100); S11(99.2)]	0.44	0.3	0.223
		[K].KGLSADSDDASDKVSETKS.[-]	3xPhospho [S7(100); S11(100); S15(97.9)]	0.53	0.15	0.136
TGGT1_209060	Thrombospondin type 1 domain-containing protein	[R].ENSQENQNAEPGETHAETEEVESNASEKLAK.[V]	3xPhospho [S3(100); S23(98.5); S26(100)]	0.61	0.15	0.21
TGGT1_229490	Tetratricopeptide repeat-containing protein	[R].NKWPGVEEESSDDGDKEGGGPSGMR.[K]	2xPhospho [S10(100); S11(100)]	0.59	0.15	0.239
TGGT1_224850	Putative polyadenylate-binding protein	[K].EGEDSGAEEEKEEEGQKR.[E]	1xPhospho [S5(100)]	0.58	0.18	0.24
TGGT1_214270	Putative translation initiation factor IF-2	[K].DDSDDETKPAPPAK.[K]	1xPhospho [S3(100)]	0.66	0.18	0.313
TGGT1_244650	Putative eukaryotic initiation factor 5	[K].KKADSDDDSDDDGQNGK.[E]	2xPhospho [S5(100); S9(100)]	0.39	0.2	0.206
TGGT1_310170	Hypothetical protein	[R].SGVTAPGGDKDTEELASDDDSDDEAGDKDEDGETNRVPGR.[D]	2xPhospho [S17(100); S21(100)]	0.64	0.21	0.289
TGGT1_204160	GYF domain-containing protein	[K].KKGDSDSEEDASEGDLATSR.[S]	3xPhospho [S5(100); S7(100); S12(99.9)]	0.58	0.22	0.295
TGGT1_216410	Hypothetical protein	[K].IVDDITADQENDESGKSDESDADKAESR.[E]	3xPhospho [S14(100); S17(100); S20(100)]	0.58	0.27	0.274
TGGT1_264990	Hypothetical protein	[R].QEGNDKVPQSAAPSASRQQSEENK.[R]	2xPhospho [S]	0.62	0.24	0.481
TGGT1_321520	Hypothetical protein	[K].CDWDKWIDSDDEDAK.[G]	1xPhospho [S9(100)]	0.61	0.26	0.222
TGGT1_228400	WD domain, G-beta repeat-containing protein	[K].NFPTPFEPKSDDDDDEDLDELR.[S]	1xPhospho [S10(100)]	0.55	0.28	0.442
TGGT1_219150	Zinc finger, zz-type domain-containing protein	[R].LLEMNVGCHEEKESDEDDGDKK.[R]	1xPhospho [S14(100)]	0.66	0.28	0.297
TGGT1_297170	Putative 50S ribosomal protein L17	[R].RPSAAEWEESDSEEEKADSPSPYK.[M]	2xPhospho [S10(98.5); S12(98.5)]	0.61	0.34	0.265
TGGT1_216730	MCM2/3/5 family protein	[R].AAEDADEGEEIASQLQSLDLSDGSKKK.[R]	2xPhospho [S21(99.1); S24(100)]	0.51	0.36	0.391
TGGT1_304970	Cell cycle-associated protein kinase CDK, Crk1	[R].LGSGNNFDEQKHQDSFR.[F]	1xPhospho [S3(100)]	0.55	0.4	0.379
TGGT1_212770	Hypothetical protein	[R].ERSDASRGPLVEALGGVDQTGADKDEK.[S]	2xPhospho [S3(100); S6(100)]	0.65	0.42	0.462

^*a*^ Listed are proteins with phosphopeptides whose numbers were either increased or decreased in PPKL^AID^ parasites treated with auxin at all timepoints tested (1, 3, and 6 hours). For the 3- and 6-hour timepoint sample sets, phosphopeptides with FC >= 2 and FC <= 0.5 are listed. For the 1-hour timepoint sample set, phosphopeptides with FC >= 1.5 and FC <= 0.66 are listed. Phosphorylation sites in the peptides are underlined.

Interestingly, among these 29 proteins, three were identified as putative interactors and/or substrates of PPKL via TurboID: SPM1 (TGGT1_263520), TGGT1_224850, and TGGT1_204160 (Fig. 7B, Table 2). SPM1, a protein that stabilizes cortical microtubules in *Toxoplasma* (41, 42), exhibited a phosphorylation increase of greater than twofold on either S14 alone or both S14 and S18 in all three timepoint sample sets. Both TGGT1_224850 and TGGT1_204160 showed decreased phosphorylation upon PPKL depletion. Interestingly, both proteins are related to translational regulation: TGGT1_224850 is a putative polyadenylate-binding protein (PABP), while TGGT1_204160 is an EIF2A homolog containing a GYF domain. These findings suggest a potential association between PPKL and translational regulation. Remarkably, S1326 of the cell cycle-related kinase Crk1 (TGGT1_304970) showed a significant decrease in phosphorylation at all three timepoints (Fig. 7B, Table 2). Crk1 has been shown to play an essential role in daughter parasite assembly (43). Thus, it is plausible that the decreased phosphorylation of S1326 in Crk1 in the absence of PPKL may contribute to the severe defect in the formation of daughter parasites in PPKL-depleted parasites.

## DISCUSSION

In this study, we conducted a comprehensive investigation into the localization and function of PPKL in *Toxoplasma*. Our findings demonstrated that PPKL localization is closely associated with the formation of daughter parasites and that depletion of PPKL has a profound impact on the initiation and development of daughter parasites, highlighting the critical regulatory role of PPKL throughout the process of daughter parasite formation.

*Toxoplasma* adopts a unique division process called endodyogeny, where two-daughter parasites are generated within the mother cell (35). The assembly of daughter parasites initiates during the late S phase of the cell cycle (35, 44). Each daughter cell is assembled around a centrosome that has already completed the replication process during the early S phase (35, 44). The exact composition of the initially assembled daughter parasites is unclear. Previous studies have identified a number of proteins that are present early in nascent daughter parasites, including FBXO1 (45), the AC9-AC10-ERK7 complex (46), the apical cap protein ISP1 (47), and the IMC proteins IMC15, IMC29, and IMC32 (27, 48, 49). In the present study, PPKL was observed as an additional early protein emerging in nascent parasites. By utilizing U-ExM, we were able to observe newly initiated daughter parasites comprising only two immature features: a potentially preconoidal region labeled by PPKL and a presumed apical polar ring labeled by an anti-acetylated tubulin antibody (Fig. 2E). This discovery suggests that PPKL appears in daughter parasites at an exceptionally early stage, precisely when the initiation of daughter parasite formation commences, and that the structures of the daughter parasite bud that were first assembled were probably the preconoidal region and the apical polar ring.

The mechanism that regulates the initiation of daughter parasite assembly also remains enigmatic. One of the prominent phenotypes observed upon PPKL depletion in our study was a significant impairment in the initiation of daughter parasites. After an 18-hour culture period, a substantial portion of PPKL-deficient parasites failed to develop detectable daughter parasites. Furthermore, when PPKL-depleted parasites were subjected to a brief 6-hour treatment with IAA, most of the dividing parasites exhibited only a single-daughter parasite. These findings indicate that the absence of PPKL disrupts the regulatory pathway responsible for initiating daughter parasite formation. Previous studies have highlighted the requirement of centrosome replication for proper cell division in *Toxoplasma*, with NEK1, MAPK2, and MAPKL1 identified as protein kinases involved in regulating centrosome duplication (36, 37, 50). Surprisingly, our investigation revealed that centrosome duplication remained unaffected in PPKL-depleted parasites. Additionally, our phosphoproteomics analysis did not indicate any impact of PPKL depletion on the phosphorylation status of NEK1 and MAPKL1. It is important to note that while certain residues in MAPK2 showed slight upregulation or downregulation, these changes were not consistently observed across all three timepoint samples. These findings indicate that PPKL potentially participates in a distinct pathway, separate from the ones responsible for regulating centrosome replication associated with NEK1, MAPK2, and MAPKL1.

Interestingly, our phosphoproteomics analysis revealed that the depletion of PPKL promptly leads to a reduction in the phosphorylation level of Crk1 at S1326, strongly suggesting that PPKL plays a role in regulating the phosphorylation state of S1326 in Crk1. In *Toxoplasma*, Crk1 is an essential cell cycle-associated kinase that partners with the cyclin protein CycL (43). Although the functional role of the Crk1-CycL complex is not well understood, conditional knockdown of Crk1 results in abnormal assembly of the daughter parasite cytoskeleton (43), suggesting that it is involved in regulating the assembly of the daughter parasite scaffold. In mammals, most cyclin-dependent kinases (CDKs) are known to be activated in a two-step process with the binding of a specific cyclin and the phosphorylation of the activation loop (T-loop) in the kinase domain, typically at a conserved threonine residue (51). Although the T-loop is present in Crk1, no phosphorylation of this conserved threonine has been reported based on available post-translational modification data of Crk1 (ToxoDB). Notably, S1326, which is ~30 aa away from the kinase domain, represents the sole identified phosphorylation site within the C-terminal region, encompassing the entire kinase domain (993–1,292 aa) and the C-terminal extension (1,293–1,373 aa), indicating that it may be a critical residue for regulating the activity of the kinase domain.

Besides the defect in daughter cell assembly, loss of PPKL resulted in structural changes to cortical microtubules. This finding is consistent with a previous study conducted on *Plasmodium*, which demonstrated that the deletion of PPKL in the parasite led to the dissociation of apical microtubules from the IMC (25). Cortical microtubules play a crucial role in maintaining the shape and stability of apicomplexan parasites (52). The normal cortical microtubule architecture of *Toxoplasma* has robust stability and can withstand strong detergent extraction (42). Our experiments using sodium deoxycholate revealed that PPKL-depleted parasites have more fragile microtubules, suggesting that PPKL is associated with the regulation of the stability of cortical microtubules. Along with the loss of stability, the cortical microtubules of PPKL-depleted daughter parasites also lose their compact and ordered structure. In those PPKL-depleted parasites undergoing their first cell division, we found that most of those single abnormal daughter parasites had a round shape with much larger gaps between microtubules. This may not only be because these single-daughter parasites are not spatially restricted in the mother parasites without a sibling but also may be mainly due to the loss of the compact and ordered structure of their cortical microtubules. In addition, we also see many other types of irregularly shaped daughter parasites in PPKL-depleted parasites, further supporting that PPKL regulates the ordered structure maintenance of cortical microtubules.

The identification of SPM1 as a putative substrate of PPKL might reveal a mechanism by which PPKL regulates microtubules. SPM1 is a filamentous microtubule inner protein, which binds to α- and β-tubulins throughout the whole length of the microtubule (41, 42). In addition to SPM1, there are two globular microtubule inner proteins in *Toxoplasma*, TrxL1 and TrxL2, bound to the microtubules (42, 53). Previous detergent extraction experiments have shown that SPM1 plays a more important role in stabilizing microtubules than both TrxL1 and TrxL2, as microtubules without SPM1 cannot withstand even mild detergent extraction, while microtubules without either TrxL1 or TrxL2 can still withstand strong detergent extraction (42). Our data suggest that PPKL may regulate SPM1 by dephosphorylating two residues, S14 and S18. Further studies are needed to verify that changes in the phosphorylation state of these two residues have a significant effect on the stability of the microtubule.

PPKL exhibits an intriguing localization pattern in both mother and daughter cells of *Toxoplasma*, specifically in the preconoidal region. This localization suggests that PPKL may have an undiscovered role in this particular region. Our ongoing research aims to elucidate the specific function of PPKL in the preconoidal region. Furthermore, we have observed that PPKL localizes to the basal complex ring of daughter parasites during the late stages of division (Fig. 2F), and the basal complex protein BCC7 is one of its neighboring proteins (Table 1), indicating a potential functional role of PPKL in the basal complex. However, previous proximity-based biotinylation labeling by multiple basal complex proteins did not identify PPKL (54). Thus, it is likely that PPKL is associated with the microtubule ends but not the basal complex itself. Accordingly, its role there may be related to the termination of the extension of microtubules since, as stated above, we have found that many PPKL-depleted parasites have elongated microtubules that run the full length of the parasite.

Both Co-IP and TurboID experiments identified DYRK1, thereby indicating a potential functional relationship between PPKL and DYRK1. Deletion of the *DYRK1* gene resulted in a slight division deficiency, suggesting a likelihood that PPKL and DYRK1 participate in a signaling pathway that regulates parasite division. The closest homolog of PPKL in plants, BSU1, positively regulates the brassinosteroid signaling pathway through the dephosphorylation of a conserved tyrosine in the CMGC family protein kinase BIN2, leading to its inactivation and degradation (10). Interestingly, *Toxoplasma* encodes for a BIN2 homolog (TGGT1_265330) that is known to be phosphorylated at the corresponding tyrosine. Nonetheless, we did not identify BIN2 as either an interactor or a putative substrate in our unbiased approaches. Interestingly, DYRK1, which also belongs to the CMGC kinase family, possesses a phosphorylated tyrosine within a conserved region similar to the phosphorylated tyrosine region of BIN2. This intriguing observation suggests the possibility that PPKL may inactivate DYRK1 by dephosphorylating this conserved tyrosine. Our ongoing research aims to investigate this potential relationship further.

All these data taken together provide a picture of the various roles potentially played by PPKL in *Toxoplasma*. Our data suggest that PPKL acts as a key regulator of daughter parasite development in *Toxoplasma*. The dynamic localization of PPKL at different stages of the cell cycle would allow it to precisely regulate the formation and development of daughter parasites as division progresses. The regulation of daughter parasite development by PPKL may begin with the regulation of an unknown pathway that activates Crk1. As division begins, PPKL is recruited earliest to the formed preconoidal region, although the role it plays there is unknown. With the assembly of microtubules, PPKL appears to play a role in maintaining the stability and compact structure of cortical microtubules, probably by dephosphorylation of the microtubule-associated protein SPM1. Interestingly, at the late stage of daughter parasite development, PPKL enriches the basal complex ring. It is not known what role it plays at this stage, but our data suggest that it may regulate the length of the microtubules, as we observed that in many PPKL-depleted parasites, the microtubules extended to the very bottom of the cell (Fig. 3E, bottom).

Overall, the findings of this study highlight the key role of PPKL, a plant-like protein phosphatase, in governing the development processes of daughter parasites in *Toxoplasma*. While potential functional relationships between PPKL and Crk1, as well as PPKL and SPM1, have been identified, the precise molecular mechanisms underlying PPKL’s involvement in the various stages of daughter parasite development remain largely elusive. Further comprehensive investigations into the regulatory mechanisms related to PPKL would greatly contribute to our understanding of the initiation and subsequent development processes of daughter parasites in *Toxoplasma*. Such studies hold great promise in providing exciting prospects for the development of antiparasitic drugs.

## MATERIALS AND METHODS

### Parasite cultures

*Toxoplasma* tachyzoites used in this study were maintained in human foreskin fibroblasts (HFFs) with standard growth medium as previously described (55). Wild-type parasites used in this study include the strain RH lacking HXGPRT and Ku80 (RH*∆ku80∆hxgprt*, referred to as *∆ku80*) (56) and the strain stably expressing the plant auxin receptor transport inhibitor response 1 (TIR1) (RH*∆ku80∆hxgprt*::TIR1, referred to as TIR1) (34).

### Generation of parasite lines

All primers used for molecular cloning and site-directed mutagenesis are listed in Supplemental Data Set S5. To tag endogenous genes with a HA or Myc epitope tag in the C-terminus, we amplified 3xHA/Myc-DHFR/HXGPRT amplicons from the plasmids LIC-3xHA-DHFR/HXGPRT or LIC-3xMyc-DHFR/HXGPRT with primers containing a 5′ overhang identical to the sequence immediately upstream of the stop codon and a 3′ overhang identical to the sequence after the Cas9 cutting site. To direct these templates to the desired locus, we generated CRISPR/Cas9 vectors by mutating the UPRT guide RNA sequence in the plasmid pSag1-Cas9-U6-sgUPRT (34) to a guide RNA sequence of the target gene by using a Q5 Site-Directed Mutagenesis Kit (NEB). The CRISPR/Cas9 plasmid and the PCR amplicon were transfected into corresponding parental parasites by using the Lonza Nucleofector and the manufacturer’s suggested protocols. Transfected parasites were selected with either pyrimethamine (for selection of the DHFR cassette) or MPA/xanthine (for selection of the HXGPRT cassette) and then cloned by limiting dilution as previously described (57).

The PPKL conditional knockdown parasite line was generated based on the same CRISPR/Cas9-mediated strategy described above. The amplicon with regions of homology with the PPKL locus was amplified from the plasmid pAID-3xHA-DHFR (34). The same CRISPR/Cas9 plasmid used for PPKL endogenous 3′ tagging was transfected together with the AID-3xHA-DHFR amplicon into the TIR1-expressing parasites (34). DYRK1 knockout parasite strains were generated based on the CRISPR/Cas9-mediated strategy described previously (57). Briefly, two guide RNA target sites were separately selected in the first exon, and the 3′ untranslated region (UTR) of the DYRK1 gene and two CRISPR/Cas9 plasmids were generated. The guide RNA expression cassette from one plasmid was amplified and inserted into the KpnI cutting site of the other plasmid to generate a CRISPR/Cas9 plasmid that expresses two guide RNAs. An amplicon of the DHFR selection cassette was co-transfected into ∆*ku80* parasites together with the double-guide RNA CRISPR/Cas9 plasmid to bridge the two cut sites via homologous recombination.

### Plaque assays

Standard plaque assays were performed as described before (55). Briefly, 500 parasites of each parasite strain were seeded into host cell monolayers grown in 12-well plates, and cultures were then grown for 6 days. Cultures were then fixed and stained with crystal violet. Host cell plaques were quantified as previously described (55).

### Immunofluorescence assays

IFAs were performed as previously described (55). The primary antibodies used included rabbit/mouse anti-HA/Myc (Cell Signaling Technology), rabbit anti-IMC6 (1:1,000), rabbit anti-centrin 1 (1:1,000), rat anti-IMC3 (1:1,000), rabbit/mouse anti-acetylated tubulin (1:5,000), and rabbit anti-*Toxoplasma* β-tubulin (1:2,000). A Nikon Eclipse E100080i microscope was used for imaging.

### Ultrastructure expansion microscopy

U-ExM was performed with intracellular and extracellular parasites as previously described (31, 32). The antibodies used included rabbit anti-HA (1:500) and anti-centrin 1 (1:500) and mouse anti-acetylated tubulin (1:500). The fluorescent antibodies used included Alexa Fluor 405 NHS-ester (1:250), Alexa Fluor 594 (1:500), Alexa Fluor 488 (1:500), and DRAQ5 Fluorescent Probe (1:250). LSM 800 and 900 microscopes were used for imaging using previously described parameters (32).

### Western blots

Western blots were performed as described previously (55). The primary antibodies used included rabbit anti-HA, anti-eIF2, anti-histone H3, and mouse anti-Sag1. The secondary antibodies utilized were HRP-labeled anti-mouse and anti-rabbit IgG. The primary antibodies were used at a dilution of 1:5,000, while the secondary antibodies were used at a dilution of 1:10,000.

### Sodium deoxycholate extraction

Parasite extraction using sodium deoxycholate was conducted following the previously described method (58). Briefly, PPKL^AID^ parasites were cultured in host cells for 18 hours and subsequently treated with either auxin or ETOH for 6 hours. Intracellular parasites were then released using syringe lysis, and the parasites were deposited onto poly-L-lysine-coated coverslips by centrifugation at 100 × *g* for 1 minute. Subsequently, the parasites were exposed to 10 mM sodium deoxycholate for 20 minutes at room temperature. Afterward, the parasites were fixed using cold methanol for 8 minutes, followed by IFA utilizing anti-acetylated tubulin and anti-IMC6 antibodies.

### Crosslinking and immunoprecipitation

Crosslinking and immunoprecipitation were performed as previously described (57) with some modifications. Briefly, intracellular parasites (PPKL-3xHA or the parental Δ*ku80* strain) grown in host cells for 24–28 hours were harvested together with host cells by scraping in cold PBS. After centrifugation, the pellet was resuspended in PBS supplemented with 5 mM DSSO (Thermo Scientific) and incubated at room temperature for 10 minutes. Crosslinking was quenched by adding Tris buffer (1 M, pH 8.0) to a final concentration of 20 mM. After two washes with PBS, the samples were lysed with 1 mL RIPA lysis buffer supplemented with protease and phosphatase inhibitor cocktail (Thermo Scientific) at 4°C for 1 hour. The lysate was then incubated with mouse IgG magnetic beads overnight at 4°C for pre-clearing and then incubated with mouse anti-HA magnetic beads for 4 hours at 4°C. After washing with RIPA lysis buffer and PBS, the beads were submitted to the Indiana University School of Medicine Proteomics Core facility for liquid chromatography coupled to tandem mass spectrometry (LC/MS-MS) analysis.

### Biotinylation by TurboID

PPKL^TurboID-HA^ or control Δ*ku80* parasites were cultured in host cells for around 24 hours. The medium was supplemented with D-biotin (dissolved in DMSO) to a final concentration of 200 µM, and cultures were incubated for 3 hours before harvesting by scraping in cold PBS. The samples were washed with cold PBS three times to eliminate biotin. Then, the samples were lysed with 1 mL cold RIPA lysis buffer supplemented with 1× protease inhibitor cocktail (Thermo Scientific) and 1 mM PMSF for 1 hour at 4°C. After centrifugation, the supernatant of the lysate was incubated with Dynabeads MyOne Streptavidin CI beads (Invitrogen) overnight at 4°C. The beads were washed two times with 1 mL of RIPA lysis buffer, once with 1 mL of 1 M KCl, once with 1 mL of 0.1 M Na_2_CO_3_, and once with 1 mL of 2 M urea in 10 mM Tris-HCl (pH 8.0) and two times with 1 mL of RIPA lysis buffer. The beads were lastly washed with PBS two times and submitted to the Indiana University School of Medicine Proteomics Core facility for LC/MS-MS.

### Phylogenetic analysis

The DYRKs used in the phylogenetic analysis included HSDYRK1A (Q13627), HSDYRK1B (Q9Y463), HSDYRK2 (Q92630), HSDYRK3 (O43781), HSDYRK4 (Q9NR20), ATDYRK1 (AT3G177500), ATDYRK2A (AT1G73460), ATDYRK2B (AT1G73450), ATDYRK3 (AT2G40120), TGDYRK1 (TGGT1_204280), and TGDYRK2 (TGGT1_283480). The sequence alignment was performed using the MUSCLE online service, the conserved regions used for tree construction were extracted by using Gblocks 0.91b, and the phylogenetic tree was constructed with a maximum likelihood method by using PhyML 3.0 with the LG model. The bootstrap values shown on the phylogenetic tree were obtained by repeating the generation of the phylogenetic tree 100 times.

### Phosphoproteomics analysis

Sample preparation, mass spectrometry analysis, bioinformatics, and data evaluation were performed in collaboration with the Center for Proteome Analysis at the Indiana University School of Medicine. The methods described below are adaptations from literature reports (59) and vendor-provided protocols.

Fifteen samples (experiment 1: *n* = 3 control, IAA 6 hours; experiment 2: *n* = 3 control, IAA 1 hour and IAA 3 hours) submitted to the Center for Proteome Analysis were denatured in 8 M urea (CHEBI: 16199) and 100 mM Tris-HCl, pH 8.5 (CHEBI: 975446756, Sigma-Aldrich, Cat No: 10812846001), with sonication using a Bioruptor sonication system (Diagenode Inc. USA, North America, Cat No: B01020001) with 30 s/30 s on/off cycles for 15 minutes in a water bath at 4°C. After subsequent centrifugation at 14,000 × *g* for 20 minutes, protein concentrations were determined by Bradford protein assay (Bio-Rad, Cat No: 5000006). Approximately 2 mg equivalent of protein from each sample was then reduced with 5 mM tris(2-carboxyethyl)phosphine hydrochloride (TCEP, Sigma-Aldrich, Cat No: C4706) for 30 minutes at room temperature and alkylated with 10 mM chloroacetamide (CAA, Sigma Aldrich, Cat No: C0267) for 30 minutes at room temperature in the dark. Samples were diluted with 50 mM Tris-HCl, pH 8.5, to a final urea concentration of 2 M for trypsin/Lys-C-based overnight protein digestion at 37°C (40 µg of protein used for global proteomics and the remainder for phosphoproteomics, 1:70 protease: substrate ratio, mass spectrometry grade, Promega Corporation, Cat No: V5072). Digestions were acidified with trifluoroacetic acid (TFA, 0.5% vol/vol) and desalted on Sep-Pak Vac cartridges (50 mg size for global and 100 mg size for phosphopeptides, Waters, Cat No: WAT054955) with a wash of 1 mL 0.1% TFA followed by elution in 70% acetonitrile and 0.1% formic acid (FA). Peptide concentrations were checked by Pierce quantitative colorimetric assay (Cat No: 23275) and confirmed to be consistent.

For phosphoproteomics, each peptide sample (approximately 2 mg) was applied to a Pierce High-Select TiO2 Phosphopeptide Enrichment Kit (Thermo Fisher Scientific, Cat No: A32993). After preparing spin tips, each sample was applied to an individual enrichment tip, washed, and eluted as per the manufacturer’s instructions. The phosphopeptide elution was immediately dried. Global peptides and phosphopeptides were each labeled with a TMT reagent (manufacturer’s instructions, 0.3 mg per global sample and 0.5 mg per phosphopeptide sample, Thermo Fisher Scientific, TMT Isobaric Label Reagent Set, Cat No: 90111 Lot XE342654 ) for 2 hours at room temperature, quenched with a final concentration vol/vol of 0.3% hydroxylamine at room temperature for 15 minutes. Labeled peptides were then mixed and dried by speed vacuum.

For high pH basic fractionation, peptides were reconstituted in 0.1% trifluoroacetic acid and fractionated on Sep-Pak Vac cartridges using methodology and reagents from the Pierce High pH reversed-phase peptide fractionation kit (eight fractions for global proteomics and four for phosphoproteomics skipping every other; Thermo Fisher Scientific, Cat No: 84868). Samples were run (one-eighth of each global and one-fifth of each phosphopeptide fraction) on an EASY-nLC 1200 HPLC system (SCR: 014993, Thermo Fisher Scientific) coupled to a Lumos Orbitrap mass spectrometer (Thermo Fisher Scientific). Peptides were separated on a 25-cm EasySpray C18 column (2 µm, 100 Å, 75 µm × 25 cm, Thermo Scientific, Cat No: ES902A) at 400 nL/minutes with a gradient of 4%–30% with mobile phase B [mobile phase A: 0.1% FA, water; mobile phase B: 0.1% FA, 80% acetonitrile (Thermo Fisher Scientific, Cat No: LS122500)] over 160 minutes, 30%–80% B over 10 minutes, and dropping from 80% to 10% B over the final 10 minutes. The mass spectrometer was operated in positive ion mode with a 4-s cycle time data-dependent acquisition method with advanced peak determination and Easy-IC (internal calibrant) on. Precursor scans (m/z 375–1,600) were done with an orbitrap resolution of 120,000, RF lens% of 30, maximum inject time of 105 ms, AGC target of 100% (4e5), MS2 intensity threshold of 2.5e4, MIPS mode, precursor filter of 70%, and 0.7 window, including charges of 2–7 for fragmentation with 30-s dynamic exclusion. MS2 scans were performed with a quadrupole isolation window of 0.7 m/z, 37% HCD CE, 50,000 resolution, 200% normalized AGC target (1e5), maximum IT of 86 ms, and fixed first mass of 100 m/z.

Raw files were analyzed in Proteome Discoverer 2.5 (Thermo Fisher Scientific) with a database containing *Toxoplasma gondii* GT1 proteins, UniProt reference *Homo sapiens* proteome, and common contaminants (total sequences: 79,215). Global and phosphoproteomics SEQUEST HT searches were conducted with a maximum number of three missed cleavages, precursor mass tolerance of 10 ppm, and fragment mass tolerance of 0.02 Da. Static modifications used for the search were (i) carbamidomethylation on cysteine (C) residues and (ii) TMT labeling on lysine (K) residues. Dynamic modifications used for the search were TMT labeling on the N-termini of peptides; oxidation of methionines; phosphorylation on serine, threonine, or tyrosine; deamidation of asparagine and glutamine; and acetylation, methionine loss, or acetylation with methionine loss on protein N-termini. The percolator false discovery rate was set to a strict setting of 0.01 and a relaxed setting of 0.05. The IMP-pm-RS node was used for all modification site localization scores. Values from both unique and razor peptides were used for quantification. In the consensus workflows, peptides were normalized by total peptide amount with no scaling. Quantification methods utilized isotopic impurity levels available from Thermo Fisher Scientific. Reporter ion quantification was allowed with an S/N threshold of 5 and a co-isolation threshold of 30%. The resulting grouped abundance values for each sample type, abundance ratio (AR) values, and respective *P* values (ANOVA) from Proteome Discoverer were exported to Microsoft Excel.

## Data Availability

The mass spectrometry data generated for this study have been deposited in the MassIVE and ProteomeXchange databases and can be accessed using the following accession numbers: (i) Co-IP: MSV000092841 and PXD045322; (ii) TurboID: MSV000092840 and PXD045321; and (iii) phosphoproteomics: MSV000092849 and PXD045354.

## SUPPLEMENTAL MATERIAL

Data Set S1 mbio.02254-23-s0001.xlsx Proteins immunoprecipitated with TgPPKL and identified by LC-MS/MS.

Data Set S2 mbio.02254-23-s0002.xlsx Proteins biotinylated by the PPKL-TurboID fusion.

Data Set S3 mbio.02254-23-s0003.xlsx Phosphopeptides identified in PPKL^AID^ parasites treated with auxin or ethanol for 6 hours by phosphoproteomics analysis.

Data Set S4 mbio.02254-23-s0004.xlsx Phosphopeptides identified in PPKL^AID^ parasites treated with auxin for 1 and 3 hours or ethanol for 1 hour.

Data Set S5 mbio.02254-23-s0005.xlsx Primers used in this study.

Figure S1 mbio.02254-23-s0006.tif PPKL localizes to the nucleus.

Figure S2 mbio.02254-23-s0007.tif Fusion of AID to the C-terminus of PPKL reduced its expression.

Figure S3 mbio.02254-23-s0008.tif PPKL^TurboID-HA^ validation.

Supplemental legends mbio.02254-23-s0009.docx Legends for Fig. S1 to S3 and Data Sets S1 to S5.

ASM does not own the copyrights to Supplemental Material that may be linked to, or accessed through, an article. The authors have granted ASM a non-exclusive, world-wide license to publish the Supplemental Material files. Please contact the corresponding author directly for reuse.
