# Supplementary figures and images for: The protein phosphatase PPKL is a key regulator of daughter parasite development in Toxoplasma gondii
Source: mBio. 2023 Oct 25;14(6):e02254-23. doi: 10.1128/mbio.02254-23 (PMC10746186; doi:10.1128/mbio.02254-23)

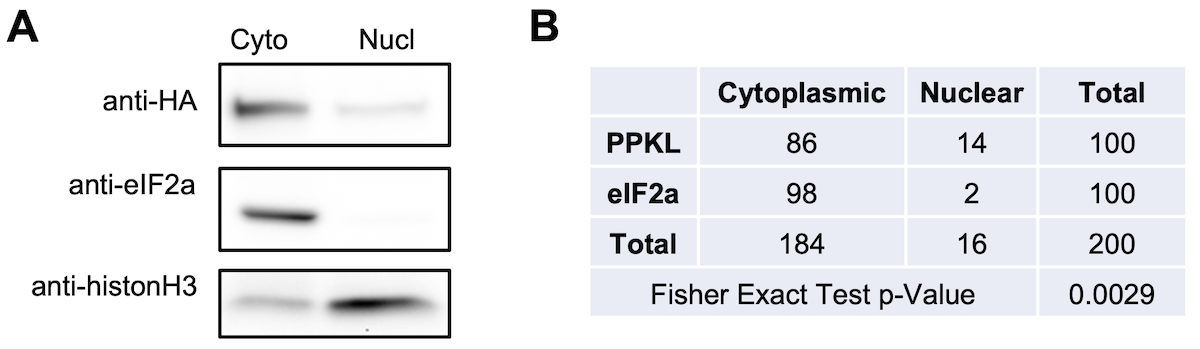

Supplement: Figure S1 — PPKL localizes to the nucleus. [file mbio.02254-23-s0006.tif]

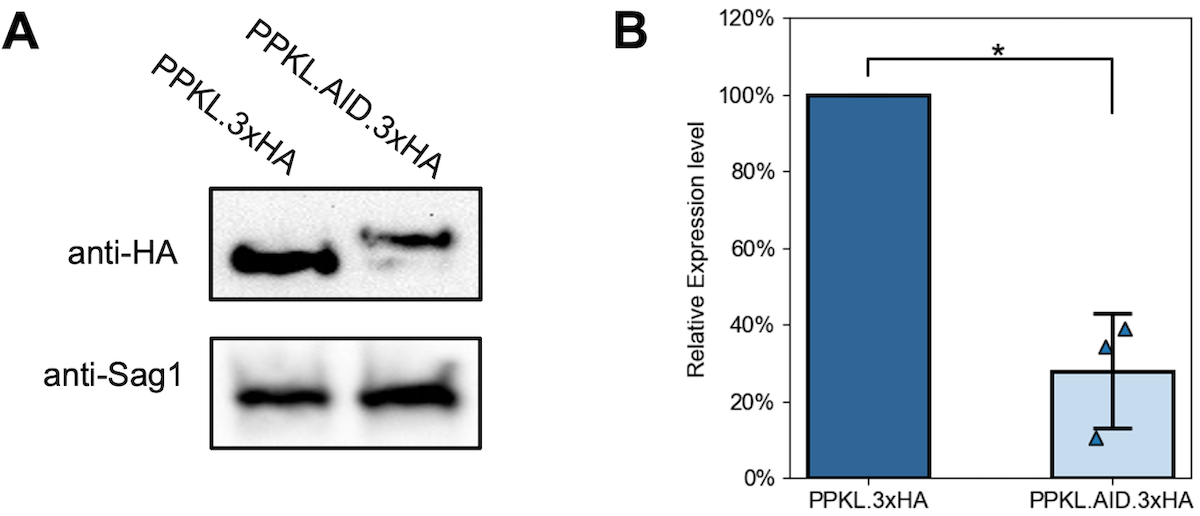

Supplement: Figure S2 — Fusion of AID to the C-terminus of PPKL reduced its expression. [file mbio.02254-23-s0007.tif]

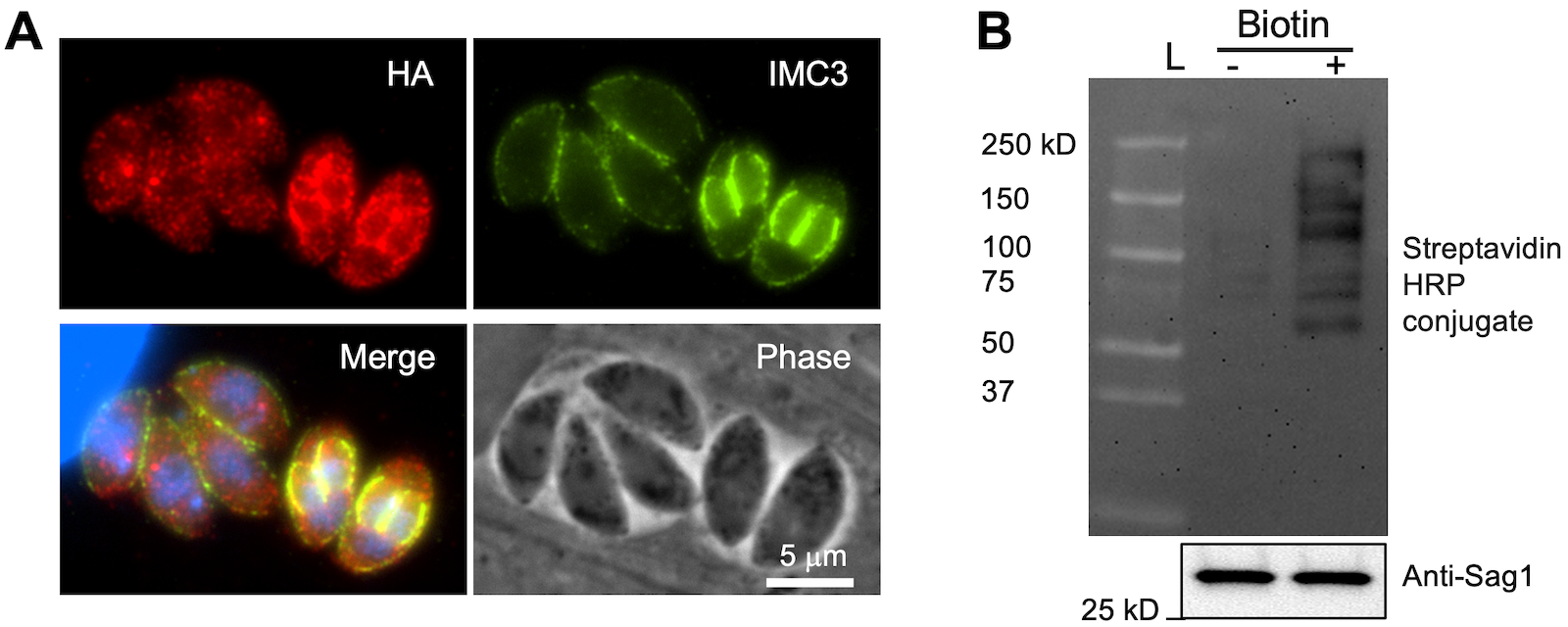

Supplement: Figure S3 — PPKLTurboID-HA validation. [file mbio.02254-23-s0008.tif]
