# Supplementary material for: The protein phosphatase PPKL is a key regulator of daughter parasite development in Toxoplasma gondii
Source: mBio. 2023 Oct 25;14(6):e02254-23. doi: 10.1128/mbio.02254-23 (PMC10746186; doi:10.1128/mbio.02254-23)
Supplement: Supplemental legends — Legends for Fig. S1 to S3 and Data Sets S1 to S5. [file mbio.02254-23-s0009.docx]

**Supplementary Figure Legends**

**Figure S1. PPKL localizes to the nucleus.** A. Western blot of protein samples after cytoplasmic and nuclear fractionation. Anti-HA was used to detect HA-tagged PPKL. Anti-eIF2α and anti-histoneH3 were used as controls to detect eIF2α, a cytoplasmic protein, and histone H3, a nuclear protein. B. ImageJ was used to quantify the relative intensity of the bands in the two portions labeled by the same antibody. Fisher exact test was used to compare the ratios of cytoplasmic/nuclear of PPKL was significantly different from that of the control eIF2α.

**Figure S2. Fusion of AID to the C-terminus of PPKL reduced its expression.** A. Western blot of protein samples isolated from PPKL^HA^ and PPKL^AID^ parasites. Anti-HA was used to detect PPKL-3xHA and PPKL-AID-3xHA. The protein Sag1 was used as a loading control. B. Quantification (mean ± std) of the Western blot of the relative expression levels of PPKL-AID-3xHA normalized to Sag1 and PPKL-3xHA. * P<0.05 (Student's t-test, two tails, unequal variance).

**Figure S3. PPKL-TurboID validation.** A. Localization of PPKL-TurboID-3xHA in intracellular parasites assessed by IFA. Scale bar: 5 μm. B. Western blot showing biotinylated proteins extracted from PPKL^TurboID^ parasites treated with or without D-biotin. Detection was achieved using Streptavidin-Conjugated Horseradish Peroxidase. Anti-Sag1 antibody was used as a loading control.

**Supplementary datasets**

**Supplementary Dataset 1.** Proteins immunoprecipitated with TgPPKL and identified by LC-MS/MS. The cutoff of fold change is PPKL.3xHA /Control >= 2.

**Supplementary Dataset 2.** List of proteins biotinylated by the PPKL-TurboID fusion. For each repeat, the fold change cutoff was PPKL-TurboID/Control >=2. The list of PPKL neighboring proteins was selected based on the following criteria: in combination with two replicates, 1) identified in both replicates; 2) p-value of Fisher Exact Test for the combined data is < 0.05.

**Supplementary Dataset 3.** Listed are phosphopeptides identified in PPKL^AID^ parasites treated with auxin or ethanol for 6 h by phosphoproteomics analysis. The sheet "PeptideGroups" contains all phospho-peptides identified in parasites and host cells. *Toxoplasma* phosphopeptides that were significantly (p-value <= 0.05) increased or decreased in auxin-treated parasites are listed in the sheets titled "Toxo Increased" and "Toxo Decreased". The phosphopeptides that were increased or decreased by more than two-fold in phosphorylation are listed in "6h Increased FC >2" and "6h Decreased FC < 0.5". The phosphopeptides that were from the proteins identified by TurboID analysis are listed in "Increase overlap with TurboID" and "Decrease overlap with TurboID". Proteins in Fig. 7A have been listed in the sheet “Proteins of Fig. 7A”. Those proteins that are PPKL neighboring proteins identified by TurboID analysis were highlighted.

**Supplementary Dataset 4.** Listed are phosphopeptides identified in PPKL^AID^ parasites treated with auxin for 1 and 3 h or ethanol for 1 h. The sheet ‘PeptideGroups’ lists all phosphopeptides identified in parasites and host cells. The phosphopeptides identified in parasites are shown in the sheet “Toxo peptides”. The phosphopeptides that are more/less abundant in 1 or 3 h auxin-treated parasites were filtered via specific fold changes and are shown in corresponding sheets. Proteins of Fig. 7A have listed in the sheet "Proteins of Fig. 7A". Proteins identified as putative PPKL neighboring proteins by TurboID are highlighted.

**Supplementary Dataset 5.** List of primers used in this study.
